# Interpretable Machine Learning for Compressive Strength Prediction of GGBS–Fly Ash Blended Concrete: Generalization Across Training-Set Compositions and Feature-Attribution Sensitivity

**DOI:** 10.3390/ma19194215

**Published:** 2026-10-03

**Authors:** Junlong Peng, Xuhang Zhu

**Affiliations:** School of Transportation, Changsha University of Science and Technology, Changsha 410114, China; pjl3168@csust.edu.cn

**Keywords:** compressive strength, GGBS–fly ash blended concrete, machine learning, training-set composition, Bayesian optimization, SHAP

## Abstract

Supplementary cementitious materials (SCMs), particularly ground granulated blast-furnace slag (GGBS) and fly ash, are widely used in concrete, but the effect of training-set SCM composition on prediction of GGBS–fly ash blended mixtures remains insufficiently examined. Six regression algorithms were compared using formulation-grouped nested cross-validation, after which the retained algorithm was optimized on the complete development set and evaluated on a formulation-disjoint hold-out set. The retained model was then examined under six training-set compositions on the same GGBS–fly ash blended hold-out subset, with additional analyses of training-set size, blended-formulation exposure, hyperparameter sensitivity, and SHAP attribution under alternative feature representations. XGBoost achieved a mean outer-fold RMSE of 5.396 MPa and a hold-out RMSE of 5.095 MPa; on the blended subset, training on all mixture types and on blended mixtures only yielded RMSEs of 5.372 and 5.346 MPa, respectively, compared with 7.095 MPa when blended formulations were excluded, while SCM SHAP attributions varied across feature representations. The retained XGBoost model showed that blended-mixture prediction performance within this dataset varied with training-set SCM composition and blended-domain representation, while SCM feature attributions varied with input representation.

## 1. Introduction

Concrete is one of the most widely used construction materials, but its extensive consumption also places considerable environmental pressure on the cement industry because ordinary Portland cement (OPC) production is energy-intensive and associated with substantial carbon emissions [1]. Reducing clinker consumption while maintaining the required engineering performance has therefore become an important route toward more sustainable concrete production [2]. Supplementary cementitious materials (SCMs) provide a practical means of partially replacing OPC, with ground granulated blast-furnace slag (GGBS) and fly ash among the most widely used alternatives [3]. The development of alternative binders and low-clinker cementitious systems has further encouraged efforts to reduce reliance on conventional Portland clinker while maintaining material performance [4,5,6]. GGBS and fly ash can be incorporated separately or in combination, resulting in concrete mixtures with different binder compositions and strength-development characteristics [7]. As these compositions become more diverse, compressive strength is governed jointly by SCM contents, water-related proportions, curing age, and other mixture variables, making it increasingly difficult to describe strength using a single predefined functional relationship [8,9].

Compressive strength is commonly established through laboratory testing, but repeated trial mixtures across broad composition ranges require considerable time and experimental effort [8,9]. Data-driven machine learning methods provide a complementary approach to strength estimation by learning relationships directly from mixture and curing records. Early studies demonstrated the applicability of artificial neural networks to concrete compressive-strength prediction [10,11], and the approach was subsequently extended to high-strength concrete [12]. Later work introduced gradient tree boosting for high-performance concrete [13], ensemble learners for sustainable high-performance mixtures [14], hybrid ensemble models [15], interpretable machine learning [16], and ensemble-learning frameworks specifically developed for high-performance concrete [17]. Together, these studies show that machine learning models can represent nonlinear relationships that are difficult to capture with conventional predefined equations and provide an efficient complement to repeated trial-based evaluation.

Machine learning applications have gradually expanded from concrete containing individual SCMs to more complex blended binder systems. Pala et al. used neural networks to examine the long-term strength effects of fly ash and silica fume [18]. Shahmansouri et al. subsequently applied gene expression programming to GGBS-based geopolymer concrete [19], while Song et al. extended machine learning prediction to fly ash-containing concrete [20]. Ahmad et al. further investigated fly ash-based geopolymer concrete using advanced machine learning techniques [21]. Machine learning approaches have also been applied to cement–fly ash–slag ternary concrete [22] and fly ash–GGBS-based geopolymer concrete [23], while broader applications have examined self-compacting concrete containing SCMs and recycled aggregate [24] and GGBFS-based geopolymer concrete using ensemble predictive models [25]. More recently, Ghrici et al. combined tree-based regression, Bayesian hyperparameter optimization, and SHAP analysis for high-performance concrete containing fly ash and blast-furnace slag, and further compared prediction errors among fly ash-only, BFS-only, and ternary mixtures [26]. Optimized XGBoost combined with SHAP has also been applied to GGBS-based geopolymer concrete [27], while explainable machine learning has been used to examine predictive behavior and feature interactions in multicomponent concrete [28]. Zhang et al. further integrated Bayesian optimization, ensemble modeling, and SHAP interpretation for slag-blended concrete [29]. These developments show that recent studies have progressed beyond prediction accuracy toward optimization, interpretability, and performance comparisons across SCM compositions. However, such studies have primarily evaluated models within a given SCM dataset or compared prediction performance among mixture categories. Less attention has been given to treating the SCM composition represented in the training data itself as a controlled analysis factor while evaluating the resulting models on the same GGBS–fly ash blended target. This distinction is important because an all-mixture training set may benefit both from broader composition coverage and from direct representation of blended formulations. The present study therefore examines how training-set SCM composition is associated with prediction performance on a common GGBS–fly ash blended target and subsequently investigates how the retained model attributes its predictions to GGBS and fly ash when both SCMs are present.

Answering these questions requires a model-development procedure that clearly separates hyperparameter selection from subsequent performance assessment. The Tree-structured Parzen Estimator (TPE) sampler implemented in Optuna provides a Bayesian approach for hyperparameter search [30]. Alongside advances in model optimization, explainable machine learning models have increasingly been applied to concrete compressive-strength prediction [31], while Bayesian optimization has been used in high-performance-concrete strength modeling [32]. Nested cross-validation further separates hyperparameter selection from evaluation on held-out outer folds, thereby reducing selection bias during development-stage performance estimation [33]. These methods were integrated with formulation-grouped data partitioning and a formulation-disjoint hold-out evaluation to establish a unified framework for candidate-model comparison, training-set composition assessment, and model interpretation.

Building on this evaluation design, six regression models are first compared using formulation-grouped nested cross-validation, and the retained model is subsequently evaluated on the formulation-disjoint hold-out set. The retained model is then trained using six different training-set compositions and evaluated against the same GGBS–fly ash blended hold-out subset. Additional sensitivity analyses are conducted to examine the influences of training-set size, blended-formulation exposure, and hyperparameter configuration on the observed performance differences. Finally, SHapley Additive exPlanations (SHAP) are used to quantify feature contributions, compare the attributions assigned to GGBS and fly ash in blended-mixture predictions, and examine the sensitivity of these attributions to alternative feature representations [34]. By linking model performance with the composition represented in the training data, the framework provides a practical basis for evaluating data coverage when machine learning models are applied to SCM-containing concrete and can support more reliable data-driven mixture assessment.

## 2. Data and Pre-Processing

### 2.1. Data Source

Data were obtained from the publicly available dataset entitled High Performance Concrete, hosted in the Mendeley Data repository [35]. The original dataset contained 1133 records, with each record comprising cement, GGBS, fly ash, water, superplasticizer, coarse aggregate, fine aggregate, curing age, and compressive strength. After data cleaning, 1107 observations were retained for subsequent analysis.

The retained records span curing ages from 1 to 365 days and cover mixtures with different GGBS and fly ash contents, providing the composition range required for the subsequent SCM-specific comparisons. The available record-level data did not provide specimen dimensions, testing standards, curing temperature or humidity, constituent properties, or laboratory and source-study identifiers.

### 2.2. Data Pre-Processing

Before model development, data pre-processing included column-name standardization, data-quality screening, manual verification, and feature construction.

Unit information, line breaks, extra spaces, and punctuation marks were removed from the original column names. The variables were then mapped to consistent names, including cement, GGBS, fly ash, water, superplasticizer, coarse aggregate, fine aggregate, curing age, and compressive strength.

The dataset was checked for missing values and duplicate records. Potentially abnormal records were screened using the Z-score and interquartile range (IQR) criteria, with thresholds of |Z| > 3 and 1.5 × IQR, respectively, followed by manual review. Twenty-five exact duplicate records and one internally inconsistent observation identified during manual review were excluded, leaving 1107 observations for subsequent analysis.

Two ratio-based features were additionally constructed: the water-to-cement ratio (W/C) and the water-to-binder ratio (W/B). They were calculated as Water/Cement and Water/(Cement + GGBS + fly ash), respectively, to represent the proportions of water relative to the cement and total binder contents.

### 2.3. Exploratory Data Analysis

Exploratory data analysis was conducted to characterize the cleaned dataset before model development. Table 1 summarizes the descriptive statistics of the eight original mixture and curing features, the two engineered ratio features, and compressive strength. The distribution of the four mixture types is also reported in Table 1.

**Figure 1:**
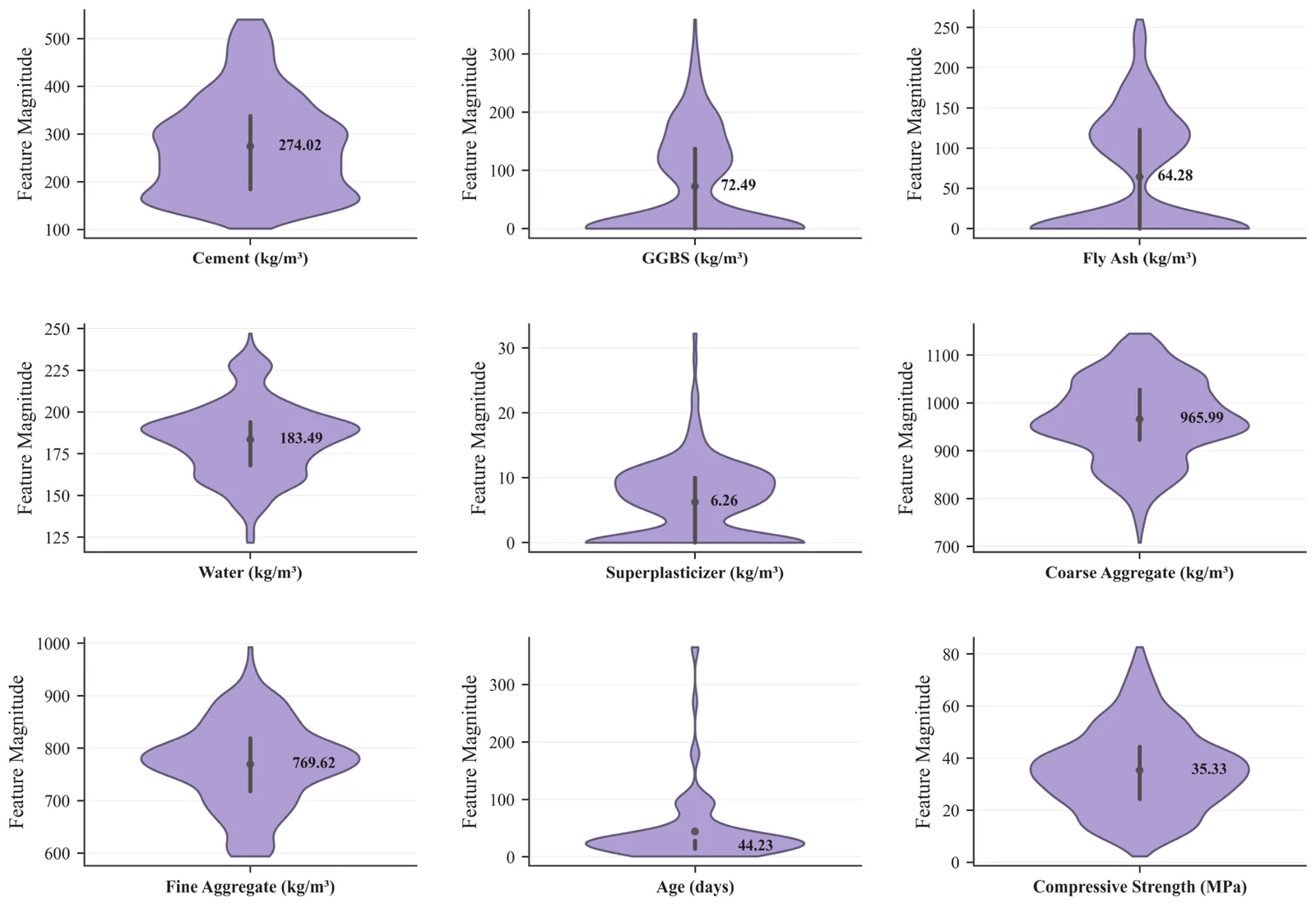
Distributions of the original mixture and curing features and compressive strength in the cleaned dataset.

The distributions of the eight original mixture and curing features and compressive strength are shown in Figure 1. Violin plots were used to show the range and distribution of each variable. These plots also show whether the samples are concentrated in a narrow interval or distributed over a wider interval. The engineered W/C and W/B features are summarized in Table 1 and were included in the subsequent correlation analysis and model development.

The samples were grouped according to the presence of GGBS and fly ash. Four mixture types were considered: GGBS-only mixtures, fly ash-only mixtures, GGBS–fly ash blended mixtures, and mixtures without either GGBS or fly ash. The sample count of each mixture type is reported in Table 1. This grouping was used to describe the SCM composition of the cleaned dataset.

**Figure 2:**
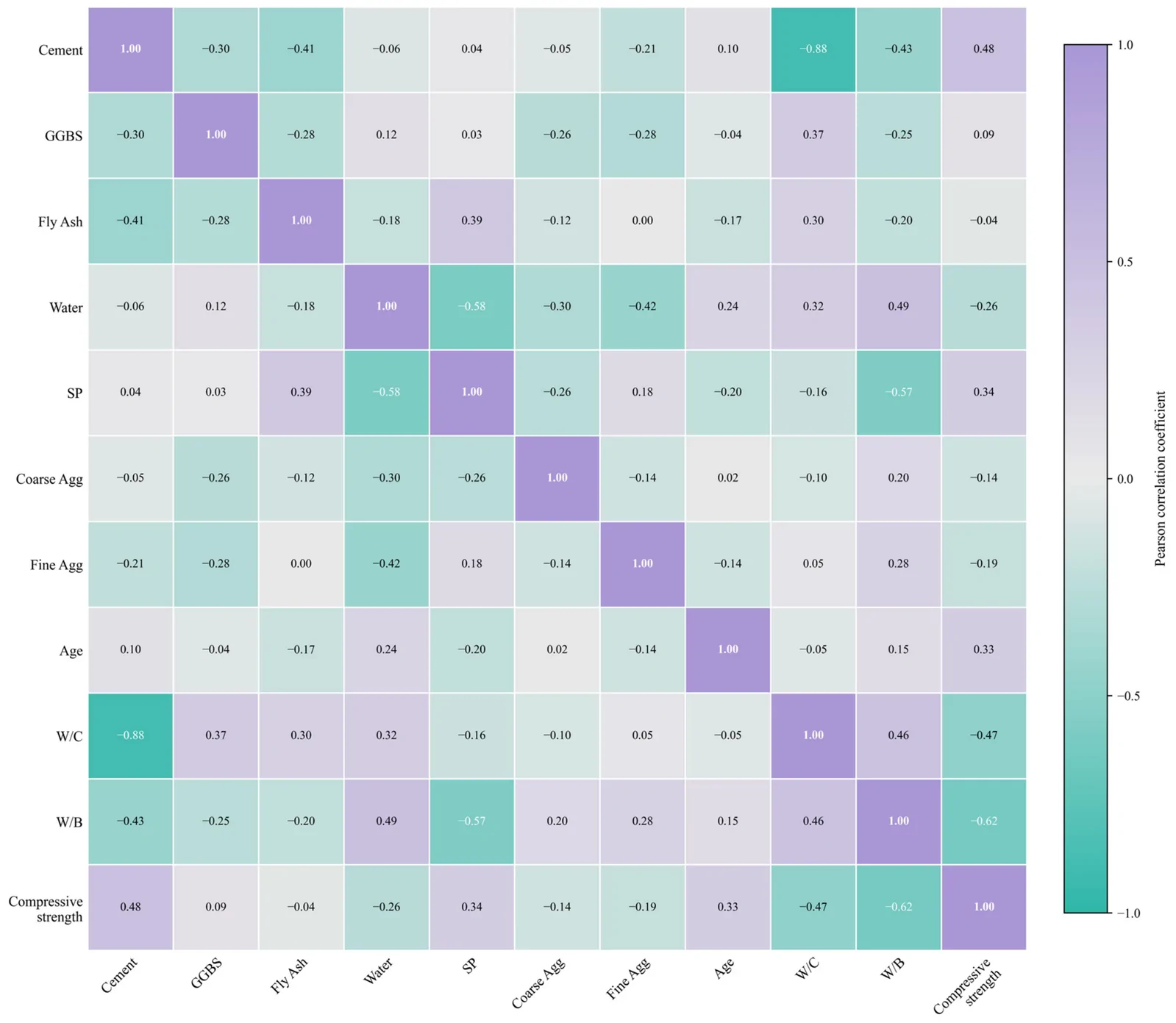
Pearson correlation matrix of the model input features and compressive strength.

Figure 2 shows the Pearson correlation matrix for the model input features and compressive strength. For most pairs of input features, the absolute correlation coefficient remained below 0.5, suggesting generally weak-to-moderate linear associations. Stronger correlations occurred for only a few feature pairs, notably Cement–W/C (r = −0.88), Water–SP (r = −0.58), and SP–W/B (r = −0.57). Among the input variables, W/B had the strongest linear correlation with compressive strength (r = −0.62), whereas the individual correlations of GGBS and fly ash with compressive strength were weak. The Pearson results were therefore used for exploratory characterization rather than as a sole criterion for removing input variables.

### 2.4. Dataset Division

The prediction of concrete compressive strength was formulated as a supervised regression task. A concrete formulation was identified using the recorded quantities of seven original mixture variables: Cement, GGBS, Fly ash, Water, SP, Coarse aggregate, and Fine aggregate. Records with identical values for these seven variables were assigned the same Formulation_ID. Age, W/C, W/B, and compressive strength were not used to define formulation identity. The 1107 cleaned observations corresponded to 530 unique formulations.

The dataset was then partitioned at the formulation level into a development set and a formulation-disjoint hold-out set. Five candidate group-aware folds were generated using stratified group splitting, with Formulation_ID used as the grouping variable and the four mixture types defined in Section 2.3 used for stratification. The hold-out fold was selected using a predefined balance criterion based on the target hold-out fraction and the mixture-type proportions at both the observation and formulation levels. Compressive strength values and model-performance information were not used in selecting the hold-out fold. The resulting development set contained 882 observations from 424 formulations, while the hold-out set contained 225 observations from 106 formulations, with no formulation overlap between the two partitions.

The development set was used for nested cross-validation, candidate-model selection, final hyperparameter determination, and construction of the training-set composition settings used in the subsequent analyses. The hold-out set remained excluded from model selection and hyperparameter optimization. After model development was completed, it was used for hold-out performance evaluation and SHAP-based interpretation. Its GGBS–fly ash blended subset, comprising 63 observations from 41 formulations, was used as the common evaluation target for the training-set composition analyses and the focused SCM attribution analysis.

### 2.5. Data Standardization

The input variables had different units and numerical ranges. Therefore, data standardization was applied before model fitting when required by the modeling pipeline.

StandardScaler was used for feature standardization in the LR, SVR, and MLP pipelines. Feature values were centered and scaled using the means and standard deviations estimated from the corresponding training data. DT, RF, and XGBoost were fitted using the original feature scales because tree-based models do not require feature standardization.

The transformation was implemented within the modeling pipeline. In each fitting process, the StandardScaler parameters were estimated from the corresponding training data. The same transformation was then applied to the corresponding validation or hold-out data.

## 3. Methodology

**Scheme 1:**
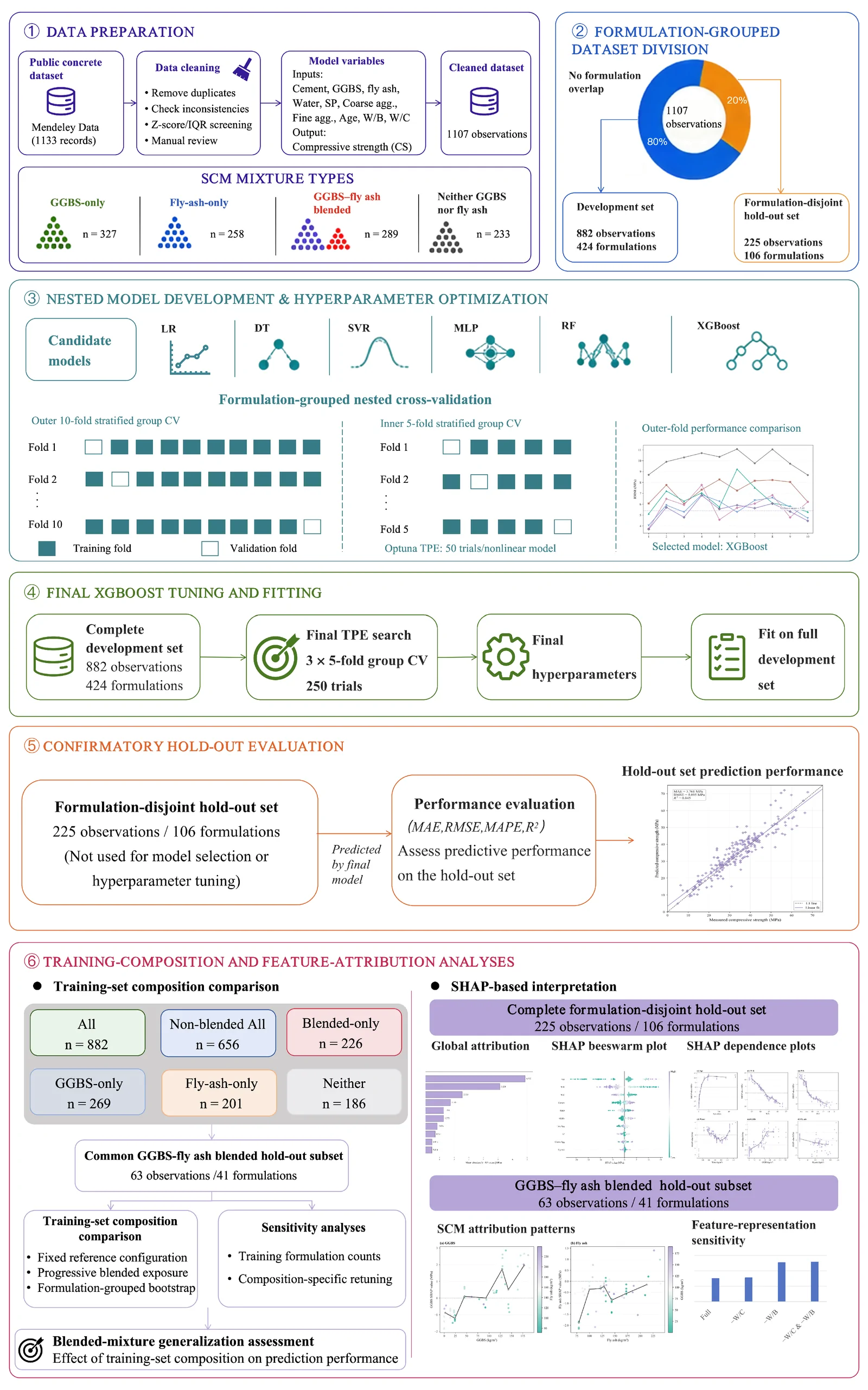
Overall workflow for model development, evaluation, and interpretation.

This section describes the methodology used to develop, evaluate, and interpret the compressive strength prediction models. The framework comprised candidate-model comparison using formulation-grouped nested cross-validation, Bayesian hyperparameter optimization, final model development, training-set composition assessment, and SHAP-based interpretation. All model-selection and hyperparameter-optimization procedures were confined to the development set. The formulation-disjoint hold-out set remained excluded from these procedures and was used only after model development for performance evaluation and subsequent interpretation. The overall methodological workflow is summarized in Scheme 1.

### 3.1. Machine Learning Models

Six supervised regression algorithms were considered to capture the linear and nonlinear relationships between mixture variables, curing age, and compressive strength: Linear Regression (LR), Decision Tree (DT), Support Vector Regression (SVR), Multilayer Perceptron (MLP), Random Forest (RF), and XGBoost. Previous studies have applied neural-network-based methods to concrete compressive strength prediction [10,11]. Tree-based boosting and ensemble-learning approaches have subsequently been used for high-performance concrete strength prediction [13,17]. These algorithms were chosen to cover complementary learning structures and to enable a balanced comparison between individual and ensemble models. LR was included as a linear reference to assess whether a linear representation was sufficient for the available data. DT, SVR, and MLP represented tree-based, kernel-based, and neural-network-based individual models, respectively. RF and XGBoost were included as tree-based ensemble models using bagging and boosting, respectively, allowing individual and ensemble models to be compared in terms of prediction accuracy and performance consistency across different data partitions.

The models were implemented in Python (version 3.10.20). Pandas (version 2.3.3) and NumPy (version 2.2.6) were used for data processing and numerical operations. LR, DT, SVR, MLP, and RF were implemented using Scikit-learn (version 1.7.2) [36], XGBoost was implemented using the XGBoost library (version 3.2.0) [37], and Optuna (version 4.8.0) was used for hyperparameter optimization [30]. Feature standardization for LR, SVR, and MLP followed the procedure described in Section 2.5 and was fitted using only the corresponding training partition.

#### 3.1.1. Linear Regression

Linear Regression (LR) models the target variable as a linear combination of the input variables, with the coefficients determined by minimizing the residual sum of squares. In this study, LR was included as a non-optimized linear reference model for comparison with the nonlinear candidate models.

#### 3.1.2. Decision Tree

Decision Tree (DT) regression recursively divides the feature space; at each node, a feature–threshold pair is selected to minimize the weighted squared error across the resulting child nodes [38]. The partitioning process continues until the predefined stopping conditions are reached, and each terminal leaf assigns the mean target value of its training samples as the prediction. Through recursive partitioning, DT can accommodate nonlinear patterns and conditional relationships among the input variables while maintaining an explicit tree structure. Tree complexity was controlled through the maximum depth, the minimum samples required for an internal-node split, the minimum samples assigned to a leaf node, and the split-selection strategy.

#### 3.1.3. Support Vector Regression

Support Vector Regression (SVR) applies the support vector machine framework to continuous-value prediction by fitting a regression function within an ε-insensitive tolerance [39]. Model complexity is controlled while deviations beyond this tolerance contribute to the optimization objective. Kernel functions allow SVR to represent nonlinear patterns in the original input space without explicitly constructing the transformed feature representation. In this study, the radial basis function (RBF) kernel was used, with the penalty coefficient C, kernel parameter γ, and ε-insensitive loss parameter ε treated as tunable hyperparameters.

#### 3.1.4. Multilayer Perceptron

The Multilayer Perceptron (MLP) used in this study consists of an input layer, one or more hidden layers, and an output layer. Nonlinear activation functions applied within the hidden layers enable the network to approximate complex relationships between the input variables and compressive strength. Model parameters are updated through backpropagation by iteratively reducing the prediction loss [40]. The MLP hyperparameters considered for optimization were the hidden-layer architecture, activation function, regularization coefficient α, initial learning rate, and batch size. MLP convergence was monitored during the nested optimization. The model was trained using the Adam optimizer, with a maximum of 5000 training epochs used for the optimization and outer-fold fitting procedures. The selected configuration that still reached this limit was subsequently refitted with the maximum increased to 20,000 epochs while retaining the previously selected hyperparameter values.

#### 3.1.5. Random Forest

Random Forest (RF) forms an ensemble of decision trees, each fitted to a bootstrap sample of the training data [41]. For each split, RF considers a randomly selected subset of input variables, which increases diversity among the individual trees through both sample and feature selection. For regression, the predictions from all trees are averaged to obtain the final output. This aggregation reduces reliance on any single-tree structure and can improve prediction stability across different samples. The RF hyperparameters considered in this study included the number of trees, maximum tree depth, minimum number of samples required to split an internal node, minimum number of samples required to be at a leaf node, and the maximum number or proportion of input variables considered at each split.

#### 3.1.6. XGBoost

XGBoost is a gradient-boosting ensemble method that constructs regression trees sequentially, with each newly added tree fitted to reduce the prediction errors remaining from the preceding ensemble [37]. The model minimizes a regularized objective function that combines the training loss with penalties on tree complexity. This sequential learning structure enables XGBoost to represent nonlinear relationships and feature interactions, while shrinkage, row and column subsampling, and regularization constrain the contribution and complexity of the individual trees.

### 3.2. Cross-Validation Strategy

Following the dataset division described in Section 2.4, all cross-validation and model-development procedures were confined to the development set. Both the outer and inner cross-validation partitions were grouped by Formulation_ID so that all observations belonging to the same concrete formulation remained within the same training or validation partition. Stratification was based on the four mixture types defined in Section 2.3. Identical outer-fold partitions were used across all six candidate models, and the same inner-fold partitions were used for the nonlinear models within each outer iteration.

Nested cross-validation was used during model development to separate hyperparameter-selection from evaluation on the held-out outer folds [33]. The outer loop comprised ten folds. In each iteration, nine folds formed the outer training subset, while the remaining fold was reserved as the outer validation subset. Hyperparameters for each nonlinear model were selected through five-fold cross-validation conducted only within the corresponding outer training subset. The selected hyperparameters were then used to refit the model on the complete outer training subset, and predictive performance was evaluated on the corresponding held-out outer fold. LR was fitted and evaluated on the same outer-fold partitions without hyperparameter optimization.

The candidate algorithms were compared using their predictive performance on the ten held-out outer folds. Mean outer-fold RMSE was used as the predefined model-selection criterion, and the algorithm with the lowest value was retained for the subsequent analyses. Fold-wise performance was also examined to characterize variation across the common outer partitions. In addition, predictions from the held-out outer folds were pooled to provide a sample-level comparison between measured and predicted compressive strengths.

After algorithm selection, a separate development-set hyperparameter optimization was conducted for the retained algorithm, as described in Section 3.3. The resulting hyperparameter values were fixed before the final model was fitted to the complete development set. The fitted model was then evaluated once on the formulation-disjoint hold-out set, which remained excluded from model selection and hyperparameter optimization.

### 3.3. Bayesian Optimization

Bayesian hyperparameter optimization was performed using the Tree-structured Parzen Estimator (TPE) sampler implemented in Optuna [30]. TPE uses the objective values obtained from completed trials to guide the search toward hyperparameter regions with a greater likelihood of improving model performance. RMSE was adopted as the optimization objective.

Hyperparameter optimization was applied to the nonlinear candidate models within the inner loop of the nested cross-validation framework described in Section 3.2, whereas LR was retained as a non-optimized linear reference. Candidate hyperparameter configurations were evaluated using only the corresponding training and validation partitions, and the configuration minimizing the validation RMSE was used for the corresponding outer-fold evaluation.

After candidate-model comparison, a separate hyperparameter optimization was conducted on the complete development set to determine the final configuration of the retained algorithm. The formulation-grouped validation principle was maintained during this procedure, and the hold-out set remained excluded from all hyperparameter-selection decisions.

### 3.4. Training-Set Composition Assessment

Six training-set compositions were constructed from the development set using the four mixture types defined in Section 2.3: All, Non-blended All, Blended-only, GGBS-only, Fly ash-only, and Neither. All included the complete development set, covering all four mixture types. Non-blended All combined the GGBS-only, Fly ash-only, and Neither categories and excluded all blended formulations. Blended-only included only formulations containing both GGBS and fly ash. GGBS-only included formulations containing GGBS but no fly ash. Fly ash-only included formulations containing fly ash but no GGBS. Neither included formulations containing neither GGBS nor fly ash. For the primary comparison, the retained algorithm was fitted separately to all available data in each setting. The input variables and the reference hyperparameter values determined on the complete development set were held fixed. All fitted models were evaluated on the same formulation-disjoint GGBS–fly ash blended hold-out subset.

Training-size sensitivity was examined by repeatedly sampling equal numbers of unique formulations from each training setting without replacement. All observations belonging to a selected formulation were retained together. Sampling was stratified by mixture type for settings containing multiple types. Observation counts and distributional differences relative to the common evaluation target were examined after sampling.

Progressive blended exposure was examined by retaining the non-blended development data and adding increasing proportions of the available blended formulations. The order of formulation inclusion varied across repetitions, with formulations selected at lower exposure levels retained at higher levels. All observations associated with the added formulations were included. Both the training-size and exposure analyses used the reference hyperparameter values from the primary comparison.

Hyperparameter sensitivity was assessed by optimizing the retained algorithm separately within each training-set composition using a common search space. Formulation-grouped nested cross-validation was conducted using only the corresponding development data. Final composition-specific hyperparameter values were then determined through grouped cross-validation using all development data available for that setting. Each model was refitted with these values and evaluated on the common blended hold-out subset for comparison with the model fitted to the same training data using the reference hyperparameter values.

### 3.5. SHAP-Based Interpretation

SHapley Additive exPlanations (SHAP) was used to interpret the predictions of the retained model [34]. The SHAP analysis was implemented using the SHAP Python package (version 0.49.1). SHAP decomposes the difference between an individual model prediction and the expected model output into contributions assigned to the input variables. The absolute SHAP value represents the magnitude of an assigned contribution, while its sign indicates the direction of that contribution.

The reference model used the final hyperparameter values determined in Section 3.3 and was fitted to the complete development set using the full feature representation. SHAP values for the complete hold-out set were used to characterize the global feature-contribution structure. The GGBS–fly ash blended subset was examined using the same fitted model to compare the contributions assigned to the two SCM variables.

Mean absolute SHAP values were calculated to rank the average contribution magnitude of the input variables. A SHAP beeswarm plot was used to examine the distribution and direction of feature contributions across individual observations. SHAP dependence plots were constructed to characterize how the contribution assigned to each selected variable varied across its observed value range. Binned median SHAP values were used to summarize the dependence patterns.

For the blended subset, the mean absolute SHAP value, mean signed SHAP value, and proportion of positive SHAP values were calculated separately for GGBS and fly ash. Pairwise SHAP interaction values were further used to quantify contributions associated with feature pairs separately from their individual main effects [42].

Feature-representation sensitivity was examined by comparing the full feature representation with three reduced representations obtained by removing W/C, W/B, or both. The seven original mixture variables and Age were retained in all four representations. The reference hyperparameter values were held fixed across these comparisons. Predictive sensitivity was assessed using repeated formulation-grouped cross-validation on the development set, with identical partitions across representations. For attribution sensitivity, a model was fitted to the complete development set for each representation. The mean absolute and mean signed SHAP values of GGBS and fly ash were then compared over the same blended hold-out subset.

### 3.6. Performance Metrics

Four metrics were used to evaluate regression performance: mean absolute error (MAE), root mean squared error (RMSE), mean absolute percentage error (MAPE), and the coefficient of determination (R^2^). MAE and RMSE quantify prediction errors on the original compressive-strength scale, whereas MAPE expresses the error relative to the measured value. R^2^ measures predictive performance relative to a mean-value reference. RMSE was also used as the objective function for hyperparameter optimization.

The mathematical expressions of the four metrics are given as follows:

(1) $$\mathrm{MAE}=\frac{1}{n}\sum_{i=1}^{n}\left|y_{i}-{\hat{y}}_{i}\right|$$

(2) $$\mathrm{RMSE}=\sqrt{\frac{1}{n}\sum_{i=1}^{n}{\left(y_{i}-{\hat{y}}_{i}\right)}^{2}}$$

(3) $$\;\mathrm{MAPE}=\frac{1}{n}\sum_{i=1}^{n}\left|\frac{y_{i}-{\hat{y}}_{i}}{y_{i}}\right|\times 100\%$$

(4) $$\;R^{2}=1-\frac{\sum_{i=1}^{n}{\left(y_{i}-{\hat{y}}_{i}\right)}^{2}}{\sum_{i=1}^{n}{\left(y_{i}-\bar{y}\right)}^{2}}$$

where *y_i_* and ${\hat{y}}_{i}$ denote the measured and predicted compressive strengths of the *i* th observation, respectively; $\bar{y}$ is the mean measured compressive strength in the corresponding evaluation set; and *n* is the number of evaluated observations.

The calculation and aggregation of the metrics depended on the evaluation procedure. For nested cross-validation, metrics were calculated separately for each held-out outer fold and summarized as means and standard deviations. Metrics based on pooled outer-fold predictions were calculated separately. For repeated cross-validation, predictions from the validation folds were pooled within each repetition before calculating the metrics, which were then summarized across repetitions. For hold-out evaluation, the metrics were calculated directly from all observations in the corresponding evaluation set.

For the training-size and progressive-exposure analyses, prediction metrics were calculated for each sampled training subset on the common evaluation target and summarized across repetitions. Differences between training settings were calculated within each repetition, and their empirical percentile ranges were used to describe variation across sampled training subsets. For distribution diagnostics, standardized mean differences were calculated as the training–target mean difference divided by the square root of the average of the two variances. Their absolute values were summarized across sampling repetitions.

Uncertainty in the common-target performance estimates and paired contrasts was assessed using formulation-grouped bootstrap resampling [43]. Formulations were sampled with replacement from the blended hold-out subset, retaining all observations associated with each sampled formulation. The same resampled formulations were used for the models being compared. Metrics and paired differences were recalculated for each resample using the existing model predictions, without refitting the models. Percentile 95% confidence intervals were obtained from the 2.5th and 97.5th percentiles of the bootstrap distributions.

## 4. Results and Discussion

This section presents the hyperparameter optimization results and compares the predictive performance of the candidate models using the held-out outer folds of formulation-grouped nested cross-validation. The selected XGBoost model is subsequently evaluated on the formulation-disjoint hold-out set after its final hyperparameter configuration has been determined using the development set.

### 4.1. Hyperparameter Optimization

Bayesian hyperparameter optimization was applied to DT, SVR, MLP, RF, and XGBoost using the Tree-structured Parzen Estimator (TPE) sampler implemented in Optuna, while LR was retained as a non-optimized linear reference. Following the formulation-grouped nested cross-validation procedure described in Section 3.2 and Section 3.3, hyperparameter optimization was conducted independently within each outer-training subset. Five-fold stratified group cross-validation was used in the inner loop, with 50 TPE trials performed for each nonlinear model in each outer iteration and mean validation RMSE adopted as the optimization objective. The hyperparameter search spaces used for the candidate models are summarized in Table 2.

Hyperparameter optimization was performed independently within each outer-training subset. The resulting optimized configurations were therefore specific to the corresponding outer iterations rather than being treated as a single fixed parameter set for each algorithm. The complete hyperparameter configurations selected in the ten outer iterations are provided in Table S1. The corresponding held-out outer-fold predictive performance is evaluated in Section 4.2.

### 4.2. Predictive Performance

The six candidate models were compared using their held-out outer-fold predictions from formulation-grouped nested cross-validation. Predictive accuracy and fold-to-fold variation were examined across the common outer partitions. The selected XGBoost model was then assessed on the formulation-disjoint hold-out set using its final development-set hyperparameter configuration.

#### 4.2.1. Cross-Validation

The six candidate models were evaluated on the same held-out outer folds of the formulation-grouped nested cross-validation. Table 3 reports the mean and standard deviation of MAE, RMSE, MAPE, and R^2^ across the ten outer folds, together with approximate 95% confidence intervals for the mean RMSE.

XGBoost achieved the lowest mean MAE, RMSE, and MAPE and the highest mean R^2^ among the six candidate models. Its mean RMSE was 5.396 MPa, followed by RF and MLP at 5.702 and 5.805 MPa, respectively. SVR, DT, and LR produced progressively larger mean prediction errors. RF showed predictive performance closest to that of XGBoost, whereas MLP exhibited greater fold-to-fold variation in RMSE.

**Figure 3:**
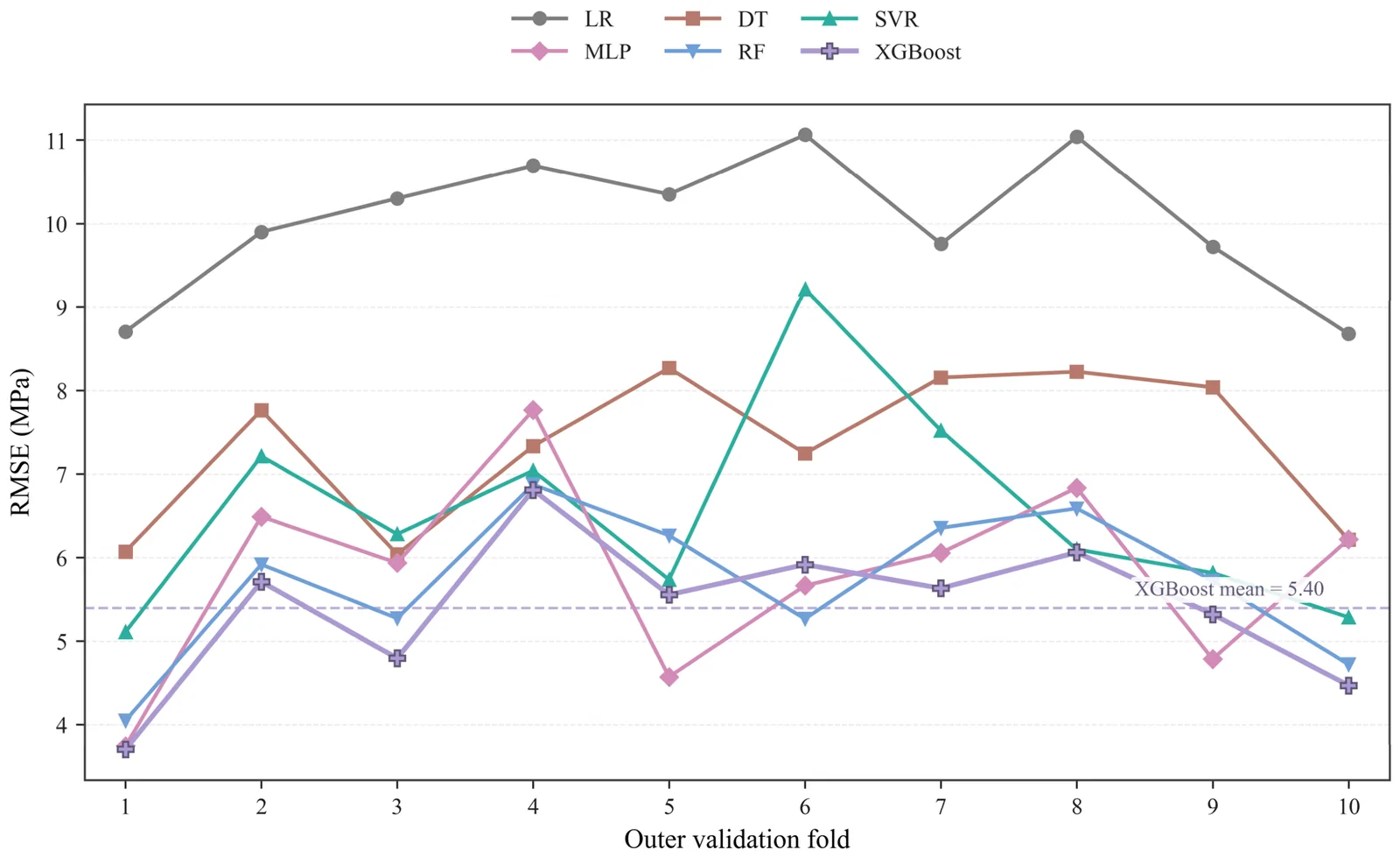
Fold-wise RMSE of the candidate models across the ten outer validation folds.

Figure 3 shows the fold-wise RMSE of the six candidate models across the ten outer validation folds. XGBoost achieved the lowest RMSE in seven of the ten folds, while MLP performed best in Folds 5 and 9 and RF in Fold 6. XGBoost and RF exhibited nearly identical fold-to-fold RMSE variability, with standard deviations of 0.882 and 0.883 MPa, respectively, whereas MLP showed greater variation with a standard deviation of 1.177 MPa.

**Figure 4:**
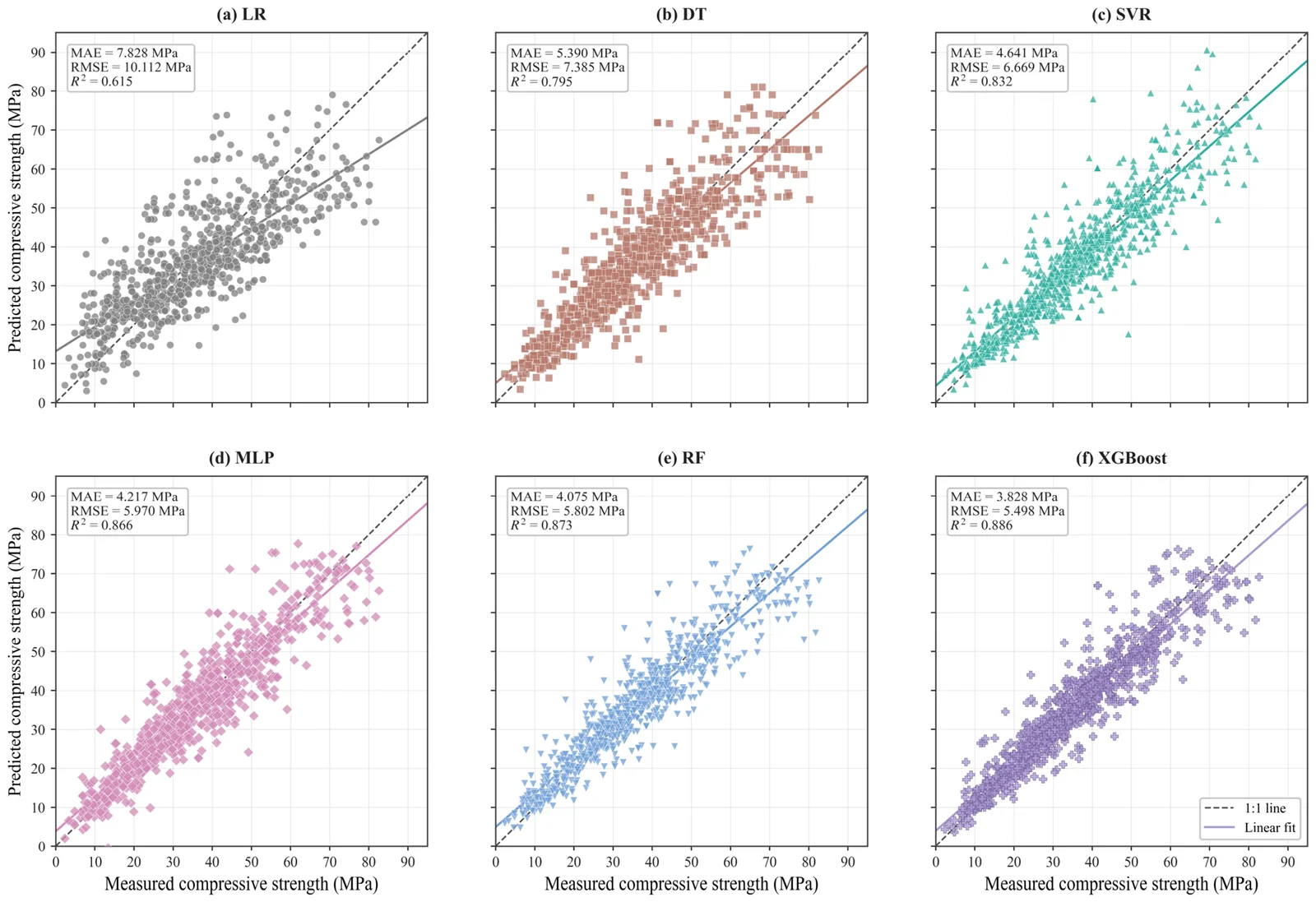
Measured and predicted compressive strengths of the candidate models based on pooled outer-fold predictions.

The pooled outer-fold predictions provide a complementary sample-level view of the candidate-model comparison. Figure 4 compares the measured and predicted compressive strengths obtained from the six models across all held-out outer-fold observations. XGBoost showed close overall agreement with the measured values, while RF and MLP exhibited similarly concentrated prediction distributions. Larger deviations were observed for SVR and DT, whereas LR showed a pronounced compression of the predicted strength range. The performance metrics shown in Figure 4 were calculated after pooling the outer-fold predictions and therefore differ slightly from the mean fold-wise metrics reported in Table 3.

Taken together, the results from Table 3 and Figure 3 and Figure 4 consistently supported the predictive performance of XGBoost among the candidate models. XGBoost achieved the lowest mean outer-fold RMSE, while RF provided the closest competing performance with nearly identical fold-to-fold RMSE variability. XGBoost also produced a lower RMSE than RF in nine of the ten common outer folds, and its pooled outer-fold predictions showed close overall agreement with the measured compressive strengths. According to the predefined model-selection criterion of the lowest mean outer-fold RMSE, XGBoost was therefore retained for the subsequent analyses.

#### 4.2.2. Hold-Out Evaluation

After XGBoost was retained through the candidate-model comparison, a separate optimization on the development set determined its final hyperparameter values. This final optimization used a search space defined separately from that used for XGBoost during candidate-model comparison. These values were fixed before the model was fitted to the complete development set and evaluated on the formulation-disjoint hold-out set. The resulting model served as the reference XGBoost model for the subsequent analyses. The hold-out set comprised 225 observations from 106 formulations, with no formulation overlap with the development set. Table 4 summarizes the resulting predictive performance.

On the formulation-disjoint hold-out set, the retained XGBoost model yielded an MAE of 3.761 MPa, an RMSE of 5.095 MPa, a MAPE of 12.609%, and an R^2^ of 0.845. The hold-out RMSE remained close to the mean outer-fold RMSE obtained during candidate-model evaluation (5.396 ± 0.882 MPa), indicating a comparable absolute prediction-error scale for previously unseen formulations.

**Figure 5:**
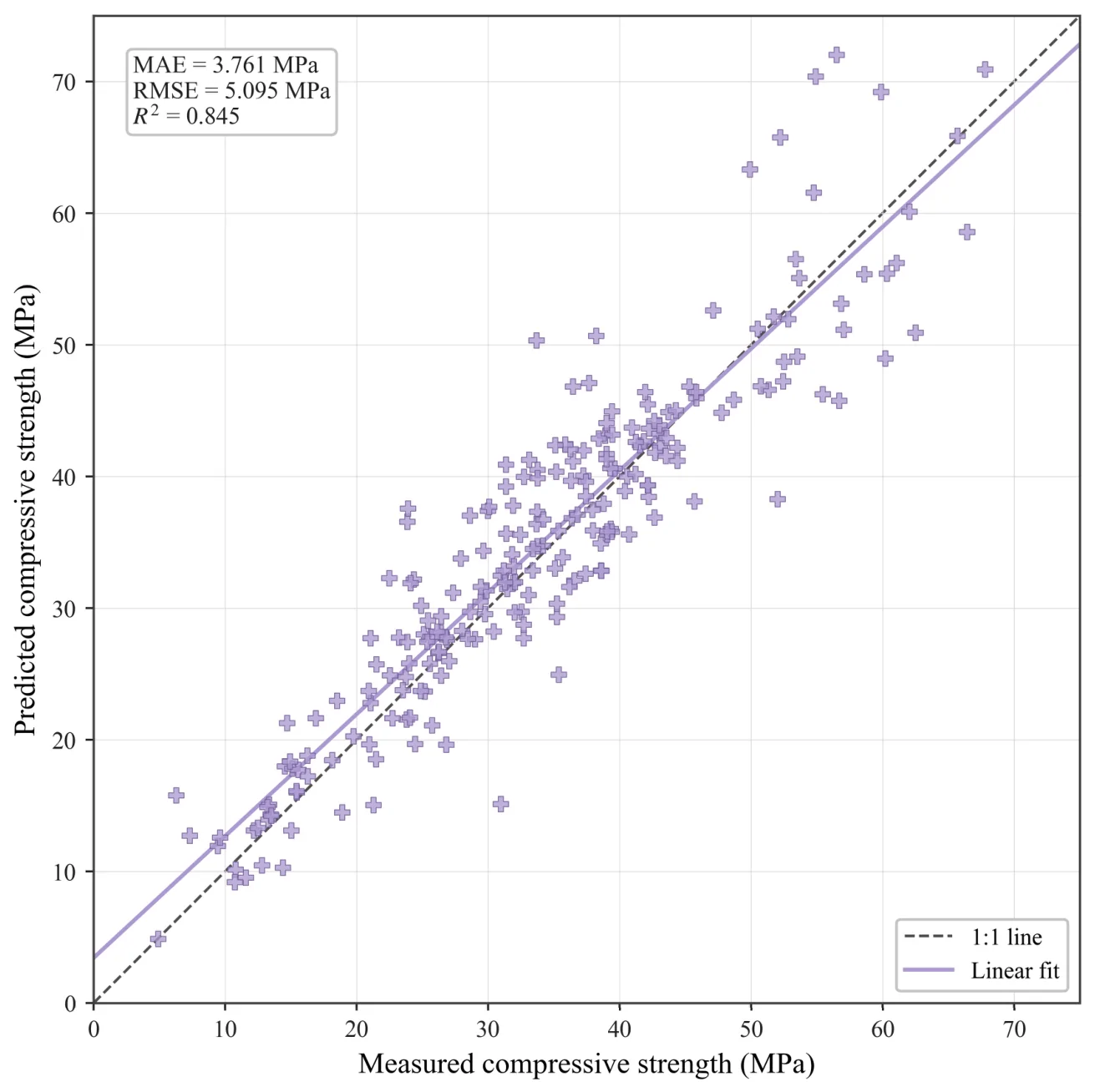
Measured and predicted compressive strengths of the retained XGBoost model on the formulation-disjoint hold-out set.

Figure 5 compares the measured and predicted compressive strengths on the hold-out set. The predictions generally followed the 1:1 reference line across the observed strength range. The mean residual, defined as measured minus predicted strength, was −0.910 MPa, while the median absolute error was 2.799 MPa. The 90th percentile of the absolute error was 8.053 MPa, indicating that larger deviations remained for a subset of observations.

The hold-out metrics and sample-level predictions indicate that the retained XGBoost model maintained a comparable absolute prediction-error level for formulations excluded from model development. This evaluation provides the basis for examining whether prediction performance for GGBS–fly ash blended mixtures varies with the SCM composition represented in the training data.

### 4.3. Model Robustness and Sensitivity

#### 4.3.1. Training-Set Composition

The retained XGBoost algorithm was fitted separately to six training-set compositions drawn from the development set: All, Non-blended All, Blended-only, GGBS-only, Fly ash-only, and Neither. The All setting included all four predefined mixture types. Non-blended All combined the GGBS-only, Fly ash-only, and Neither mixtures while excluding all GGBS–fly ash blended formulations. Blended-only, GGBS-only, and Fly ash-only contained their corresponding mixture types, whereas Neither comprised mixtures containing neither GGBS nor fly ash. In this training-composition comparison, all six models used the input variables and hyperparameter values of the reference XGBoost model evaluated in Section 4.2.2. All fitted models were evaluated on the same formulation-disjoint GGBS–fly ash blended hold-out subset, comprising 63 observations from 41 formulations. Table 5 summarizes the training-set sizes and predictive performance.

**Figure 6:**
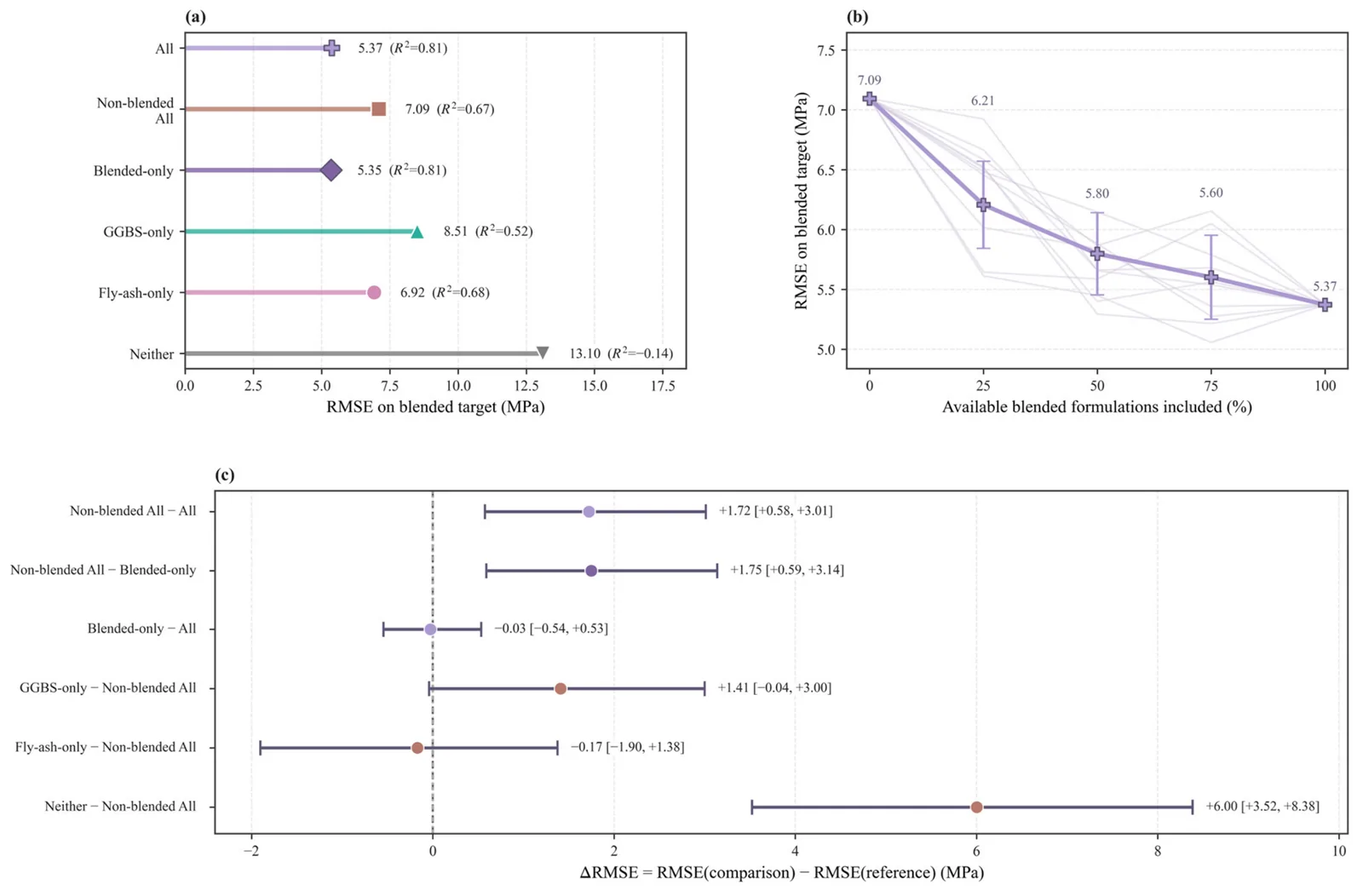
Blended-mixture prediction under different training-set compositions: (**a**) Training-set composition; (**b**) Progressive blended-domain exposure (50 repetitions; mean ± 1 SD; pale lines: 10 of 50 repetitions); (**c**) Paired formulation-grouped bootstrap contrasts (positive values favor the reference setting; 95% formulation-bootstrap intervals).

The All setting yielded a lower RMSE than Non-blended All, with values of 5.372 and 7.095 MPa, respectively (Table 5 and Figure 6a). The paired RMSE difference, calculated as Non-blended All minus All, was 1.723 MPa, with a 95% formulation-grouped bootstrap confidence interval of [0.575, 3.013] MPa (Figure 6c). The interval remained above zero, supporting lower prediction error for All on the common blended target under the evaluated settings.

The Blended-only setting achieved an RMSE of 5.346 MPa. Its comparison with Non-blended All also favored training with blended formulations: the paired difference, calculated as Non-blended All minus Blended-only, was 1.748 MPa, with a 95% bootstrap confidence interval of [0.590, 3.137] MPa. The difference between Blended-only and All was much smaller at −0.025 MPa, with an interval of [−0.544, 0.533] MPa that included zero (Figure 6c). These results did not establish an additional predictive benefit from including non-blended mixtures alongside the blended training data under the common hyperparameter configuration. At the other extreme, the Neither setting yielded the largest prediction error (RMSE = 13.098 MPa; R^2^ = −0.140). In this setting, GGBS and fly ash were absent from all training formulations, and W/B consequently coincided with W/C, whereas the blended evaluation subset contained both SCMs and a distinct W/B–W/C relationship. This pronounced training–target feature-space mismatch is consistent with the negative R^2^ and the comparatively large prediction error.

#### 4.3.2. Training Size and Exposure

Training-size sensitivity was examined using 25, 50, and 75 unique formulations from each of the six training-set compositions. All records belonging to a selected formulation were retained together, and the sampling and model-fitting procedure was repeated 50 times for each setting and size. The maximum common size of 75 formulations was determined by the available formulations in the Neither setting. A separate progressive-exposure analysis retained all 284 non-blended development formulations and successively included 0%, 25%, 50%, 75%, and 100% of the 140 available blended formulations, also using 50 repetitions. Both analyses retained the input variables and hyperparameter values used in Section 4.3.1 and evaluated the refitted models on the same blended hold-out subset.

All yielded a lower mean RMSE than Non-blended All at each common formulation count. The mean difference, calculated as Non-blended All minus All, increased from 0.454 MPa at 25 formulations to 0.606 and 0.770 MPa at 50 and 75 formulations, respectively. At 75 formulations, the corresponding mean RMSE values were 6.502 and 7.271 MPa, and All had lower RMSE in 44 of the 50 repetitions. Across these repetitions, the empirical 2.5th–97.5th percentile range of the paired RMSE differences was [−0.845, 2.760] MPa, showing that the direction and magnitude of the difference varied among sampled training subsets. Blended-only yielded a similar mean RMSE of 6.504 MPa at the same formulation count. The effect of increasing formulation count was not uniform across the training domains; for the Neither setting, the mean RMSE increased from 10.537 MPa at 25 formulations to 13.098 MPa at 75 formulations.

Equal formulation counts did not equalize the number of observations or the covariate distributions. At 75 formulations, All and Non-blended All contained an average of 153.16 and 172.22 observations, respectively. Their mean absolute standardized mean differences relative to the blended target were 0.507 and 0.720 for W/B, and 0.401 and 0.589 for total binder content. These distribution differences remained after the number of training formulations was controlled. The equal-count analysis therefore controlled formulation count but did not eliminate differences in observation count or covariate coverage, preventing the remaining prediction-error differences from being attributed solely to SCM composition.

Figure 6b shows the change in prediction error under progressive inclusion of blended training formulations. The bold line represents the mean RMSE across 50 repetitions, and the error bars indicate ±1 standard deviation. The pale lines show 10 of the 50 repetition trajectories. The mean RMSE decreased from 7.095 MPa at 0% inclusion to 6.207, 5.798, 5.601, and 5.372 MPa at 25%, 50%, 75%, and 100% inclusion, respectively. Variation across sampled training subsets remained at the intermediate levels, and individual trajectories did not consistently follow the monotonic mean trend. The 100% level reproduced the All setting, with both non-blended and blended formulations retained. The reduction in mean error accompanied an increase in total training size from 284 to 424 formulations and cannot be interpreted as the effect of blended exposure at a fixed training size.

#### 4.3.3. Hyperparameter Sensitivity

The hyperparameter values used in Section 4.3.1 and Section 4.3.2 were compared with values optimized separately for each training composition. Each composition-specific search used only the corresponding development data, with a common search space across the six compositions. Formulation-grouped nested cross-validation with five outer folds and three inner folds was used for development-stage assessment. A separate five-fold search using all available development data within each composition determined its final hyperparameter values. Each model was then fitted to its corresponding development data and evaluated on the common blended hold-out subset.

All retained a lower target RMSE than Non-blended All after composition-specific tuning, with values of 5.335 and 7.683 MPa, respectively. Blended-only also yielded a lower RMSE than Non-blended All, at 6.382 MPa. The point-estimate ordering for these two contrasts was consistent with that obtained under the common configuration.

The relative ordering of All and Blended-only changed under composition-specific tuning. The RMSE for Blended-only increased from 5.346 to 6.382 MPa, whereas that for All decreased slightly from 5.372 to 5.335 MPa. For Blended-only, the paired RMSE change, defined as RMSE (retuned)–RMSE (fixed), was 1.036 MPa, with a 95% formulation-grouped bootstrap confidence interval of [0.192, 1.839] MPa. The corresponding change intervals for the other five training settings included zero.

The sensitivity analysis supports the persistence of the primary All–Non-blended All contrast under the evaluated tuning procedure, but not a configuration-invariant ranking of all training settings. The magnitude of the target prediction error and some relative comparisons remained sensitive to hyperparameter selection. These results complement the training-size and distribution analyses without separating the effects of training-set composition, data coverage, and parameter configuration completely.

### 4.4. SHAP Interpretation

SHAP analysis was applied to the reference XGBoost model evaluated in Section 4.2.2, which was fitted to the complete development set using the full feature representation.

#### 4.4.1. Global Feature Attribution

**Figure 7:**
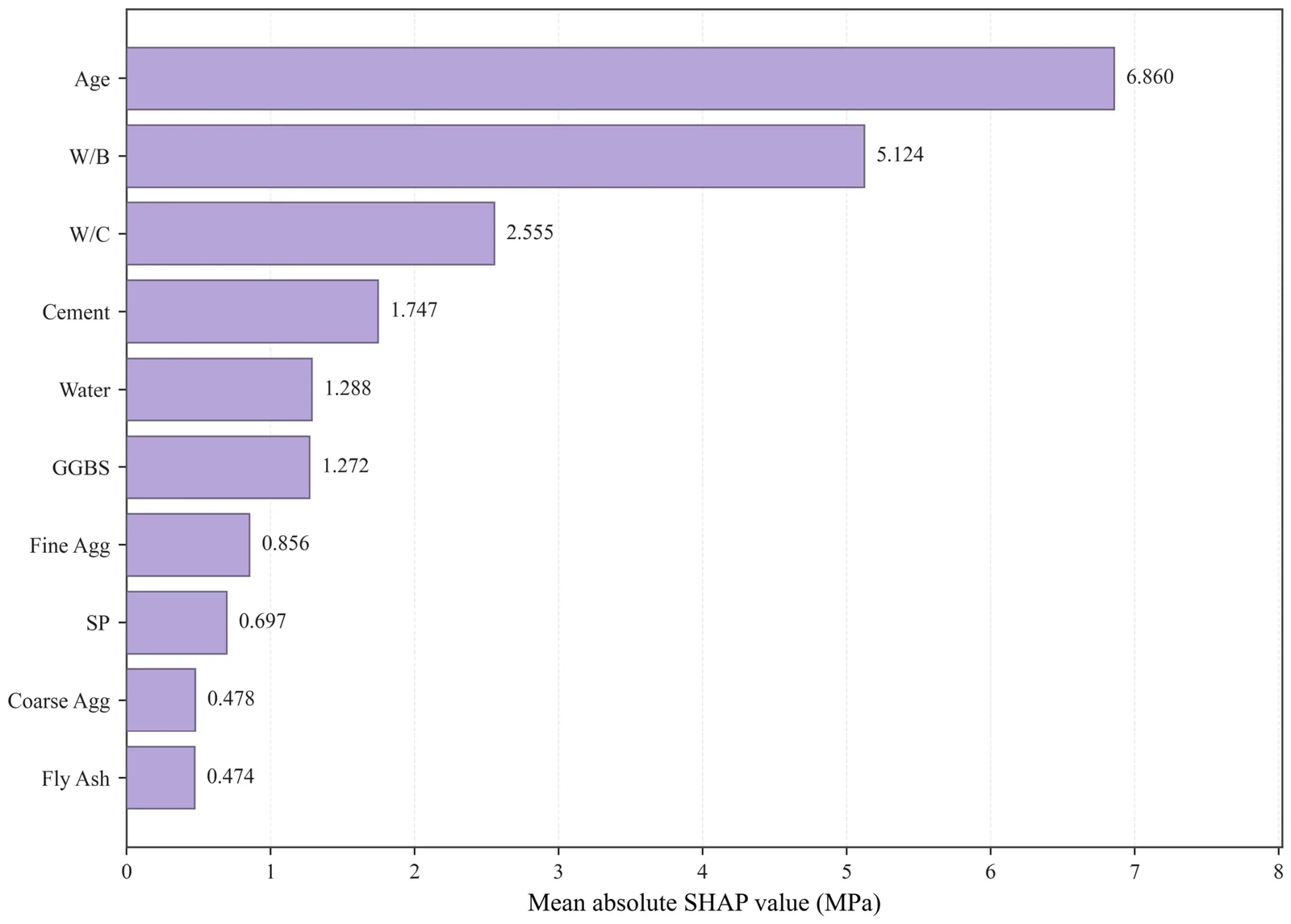
Global SHAP importance of the retained XGBoost model.

Global feature importance was summarized using the mean absolute SHAP values across the 225 observations in the complete formulation-disjoint hold-out set. As shown in Figure 7, Age had the largest mean absolute SHAP value of 6.860 MPa, followed by W/B and W/C at 5.124 and 2.555 MPa, respectively. Cement and Water ranked fourth and fifth, with values of 1.747 and 1.288 MPa. GGBS ranked sixth at 1.272 MPa, whereas fly ash had the smallest mean absolute SHAP value of 0.474 MPa. The ranking indicates that the retained model assigned larger average attribution magnitudes to Age and the water-related ratio variables than to the individual SCM contents within the full feature representation. Age and W/B have also been identified among the influential features in a recent SHAP-based analysis of GGBS-based concrete [44].

**Figure 8:**
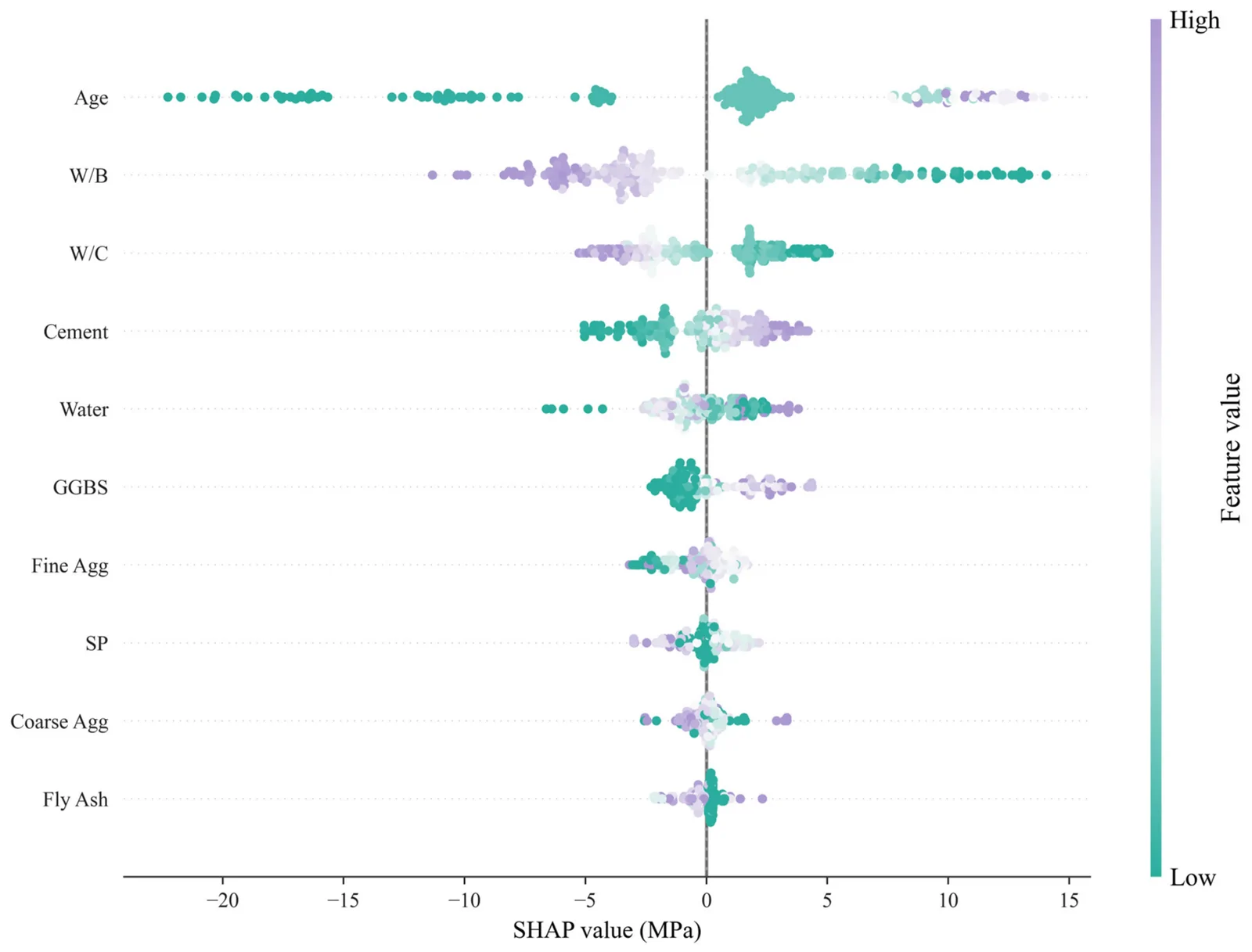
SHAP contribution distributions on the complete hold-out set.

Mean absolute SHAP values summarize contribution magnitude without showing its direction or variation across individual observations. Figure 8 presents the SHAP beeswarm plot for the complete formulation-disjoint hold-out set. Each point represents one observation for a given feature, with its horizontal position indicating the SHAP value and its color indicating the feature value, from teal for low values to purple for high values. Lower Age values were predominantly associated with negative SHAP values, whereas higher ages were associated with positive values. W/B and W/C showed the opposite pattern, with higher ratios generally associated with negative contributions. Higher Cement and GGBS contents tended to correspond to positive contributions. Fly ash showed both positive and negative contributions within a comparatively narrow SHAP range.

#### 4.4.2. Feature Dependence

**Figure 9:**
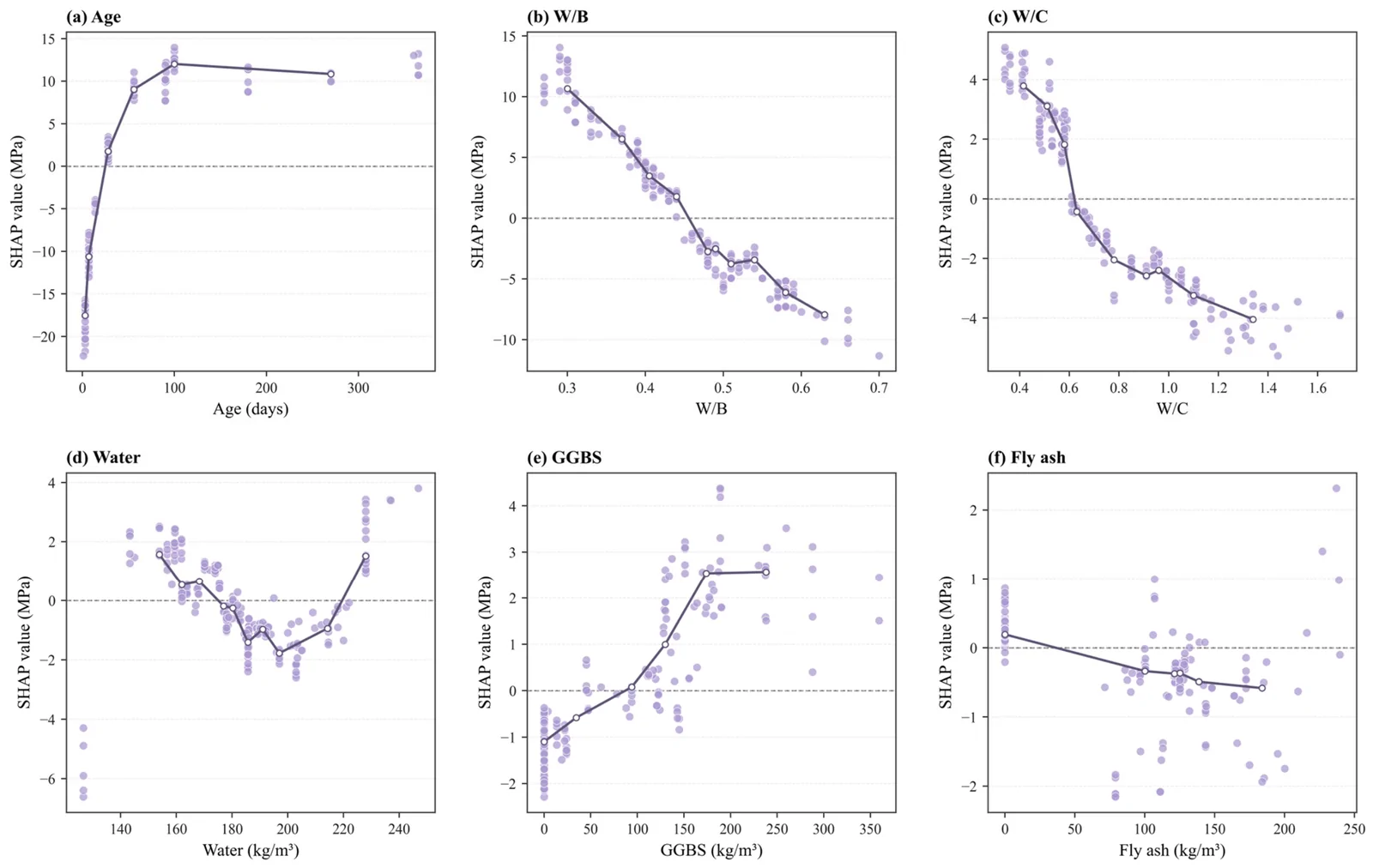
SHAP dependence patterns of the selected features.

Figure 9 extends the global attribution analysis by showing how SHAP values vary with the values of six selected features on the complete formulation-disjoint hold-out set. Age, W/B, and W/C were examined as the three highest-ranked features in Figure 7. Water was included as a common component of both ratio variables, while GGBS and fly ash were examined as the SCMs of primary interest. Each point represents an individual observation, and the solid lines connect the median SHAP values within successive feature-value bins. The horizontal dashed lines mark zero SHAP values.

Age displayed a pronounced nonlinear dependence pattern in Figure 9a. SHAP values were negative for observations at 1–14 days and positive at 28 days. The binned trend increased further towards approximately 100 days and changed less at later ages, where the contributions remained positive. The pattern shows a marked change in the attribution assigned to Age over the early-to-intermediate age range, followed by a more gradual variation among later-age observations.

W/B and W/C showed broadly decreasing SHAP patterns in Figure 9b,c. Their binned trends shifted from positive to negative values as the ratios increased, with a wider SHAP range for W/B. The decline in W/C attribution was more pronounced around the transition near 0.6 and became more gradual at higher ratios. Water exhibited a non-monotonic pattern in Figure 9d. The binned trend decreased from positive values around 150–170 kg/m^3^ to negative values around 180–210 kg/m^3^, before rising towards positive values near 230 kg/m^3^. The few observations near 125 kg/m^3^ also had negative SHAP values, distinguishing the low-content observations from the subsequent descending-and-rising trend.

GGBS contributions generally shifted from negative values at zero and low contents towards positive values at higher contents (Figure 9e). The binned median was close to zero around 100 kg/m^3^ and increased to approximately 2.5 MPa over the range of 175–240 kg/m^3^. Individual SHAP values varied at comparable GGBS contents, particularly in the intermediate range. Fly ash showed a narrower SHAP range in Figure 9f. Its binned median shifted from slightly positive values at zero content towards modestly negative values across much of the nonzero range. Both positive and negative contributions occurred among the higher-content observations, indicating variation around the central trend.

#### 4.4.3. SCM Contributions

The SHAP values of GGBS and fly ash were further summarized for the blended hold-out subset, comprising 63 observations from 41 formulations in which both SCMs were present. The analysis used the same reference XGBoost model as the full hold-out analysis. Table 6 compares the mean absolute SHAP values, mean signed SHAP values, and proportions of positive SHAP values for the two SCM variables in the complete hold-out set and the blended subset.

The mean absolute SHAP value of GGBS decreased from 1.272 MPa in the complete hold-out set to 1.125 MPa in the blended subset, whereas that of fly ash increased from 0.474 to 0.722 MPa. GGBS retained the larger average attribution magnitude, but the difference between the two SCM variables narrowed within the blended subset. Their average contribution directions also differed. GGBS had a mean signed SHAP value of +0.250 MPa, with positive contributions in 52.4% of the blended observations. Fly ash had a mean signed SHAP value of −0.603 MPa, with positive contributions in 12.7% of the observations.

**Figure 10:**
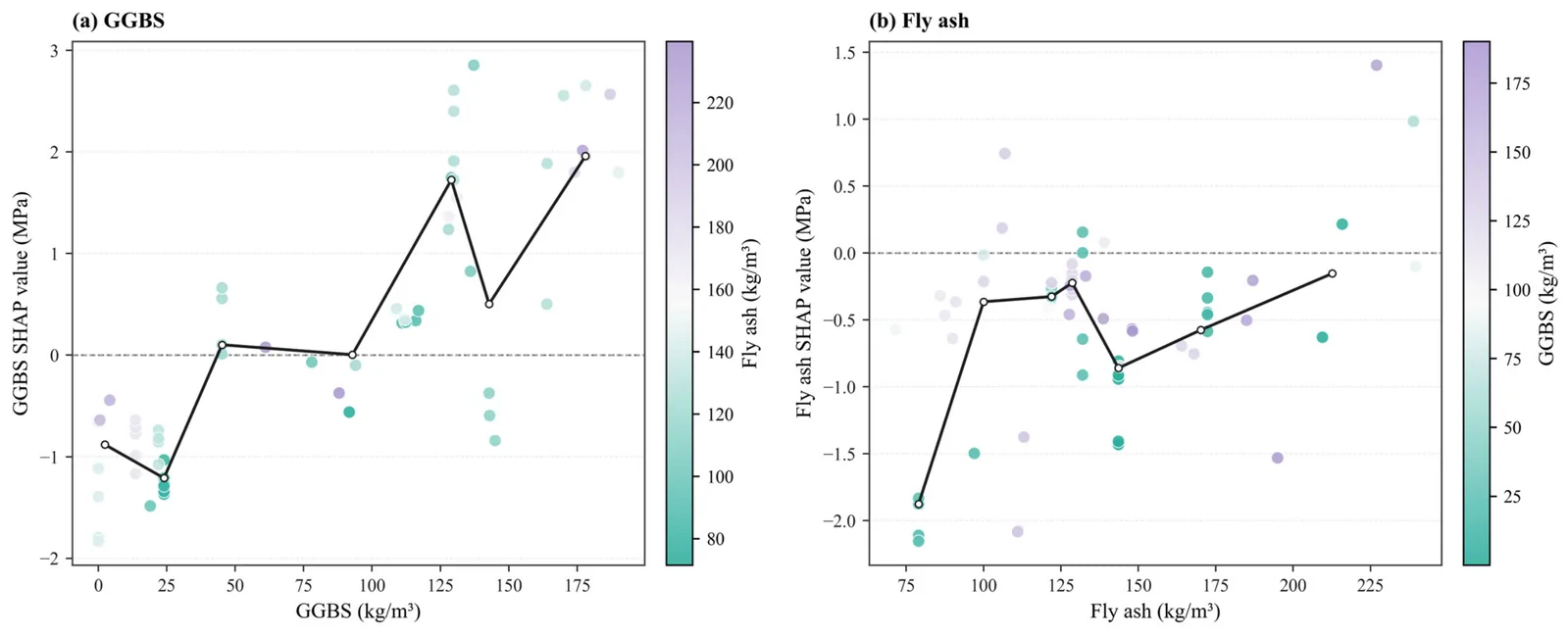
SHAP dependence patterns of GGBS and fly ash in blended mixtures.

Figure 10 shows the dependence patterns of the two SCM variables within the blended subset. Points are colored by the content of the accompanying SCM, and the solid lines connect the binned median SHAP values. GGBS contributions were predominantly negative at low contents and generally became positive at higher contents, with local fluctuations in the intermediate range (Figure 10a). The median trend rose near 130 kg/m^3^, declined around 140–145 kg/m^3^, and increased again towards the upper end of the observed range. Fly ash showed a different pattern in Figure 10b. Its binned median remained below zero across the displayed range, became less negative between approximately 80 and 130 kg/m^3^, declined again near 140 kg/m^3^, and then approached zero at higher contents. Although individual observations occurred on both sides of the zero line, most fly ash SHAP values were negative.

The mean absolute off-diagonal GGBS–fly ash SHAP interaction value was 0.035 MPa on the complete hold-out set and 0.052 MPa on the blended hold-out subset, placing this feature pair 44th and 37th among the 45 feature pairs, respectively. These values characterize pairwise attribution within the fitted model and are distinct from the prediction differences among training-set compositions examined in Section 4.3. Accordingly, the small pairwise interaction does not imply adequate coverage of the blended training domain; the higher prediction errors observed after excluding blended formulations may reflect differences in data distribution and domain coverage.

#### 4.4.4. Feature-Representation Sensitivity

To examine the sensitivity of the results to the derived ratio variables, the full feature representation was compared with three reduced representations obtained by removing W/C, W/B, or both variables. The seven original mixture variables and Age were retained in all four representations. The hyperparameter values of the reference XGBoost model evaluated in Section 4.2.2 were held fixed throughout this feature-representation comparison. Predictive sensitivity was evaluated using three repetitions of five-fold formulation-grouped cross-validation on the development set, with identical partitions across the four representations. For the SHAP comparison, a separate model was fitted to the complete development set for each feature representation, and its attributions were summarized over the same blended hold-out subset. The results are summarized in Table 7.

Removing W/C had little effect on predictive performance, with the mean RMSE changing from 5.258 to 5.275 MPa and R^2^ from 0.896 to 0.895. In contrast, removing W/B increased the mean RMSE to 5.771 MPa and reduced R^2^ to 0.875. Removing both ratio variables produced a similar result, with an RMSE of 5.788 MPa and an R^2^ of 0.874. The predictive results were therefore more sensitive to the removal of W/B than to the removal of W/C.

The SCM attribution results also varied across the alternative feature representations. Within the blended hold-out subset, the mean absolute SHAP values of GGBS and fly ash were 1.125 and 0.722 MPa under the full representation. After removing W/C, the corresponding values changed to 0.862 and 1.140 MPa, reversing their ordering by mean absolute SHAP value. Removing W/B increased the mean absolute SHAP values to 3.020 MPa for GGBS and 1.396 MPa for fly ash. The mean signed SHAP value of fly ash also changed from −0.603 MPa under the full representation to +0.924 MPa after W/B was removed. When both ratio variables were removed, the corresponding mean absolute SHAP values were 2.669 and 1.149 MPa, with positive mean signed SHAP values for both SCM variables. These results show that the magnitude and average direction of the SCM attributions varied with the inclusion of the derived ratio variables.

## 5. Conclusions

This study compared six regression algorithms for compressive-strength prediction of concrete with varying GGBS and fly ash contents under a formulation-aware model-development and evaluation framework. The retained XGBoost model was subsequently used to examine how prediction performance on GGBS–fly ash blended mixtures varied with the SCM composition represented in the training data, followed by SHAP-based analysis of feature attribution and its sensitivity to alternative feature representations. The principal conclusions are as follows.

(1) Among the six candidate algorithms, XGBoost achieved the lowest mean outer-fold RMSE of 5.396 ± 0.882 MPa and produced the lowest RMSE in seven of the ten formulation-grouped outer folds. After the algorithm had been retained and its final hyperparameters were determined using the development set, the resulting model achieved an RMSE of 5.095 MPa and an R^2^ of 0.845 on the formulation-disjoint hold-out set. The comparable prediction-error levels obtained from the outer-fold evaluation and the hold-out evaluation supported the use of XGBoost as the reference model for the subsequent training-composition and interpretation analyses.
(2) Across the primary comparison and the subsequent sensitivity analyses, models trained with representation of blended formulations showed lower mean prediction error on the common blended target than the broad training setting from which blended formulations were excluded. The principal All–Non-blended All contrast was retained after separate analyses of formulation count and hyperparameter configuration. These results identify training-set SCM composition and blended-domain representation as important considerations for generalization to GGBS–fly ash blended mixtures, while also showing that data coverage should be considered when interpreting the magnitude of the observed differences.
(3) SHAP analysis characterized the feature-attribution structure of the retained XGBoost model at both the global and blended-mixture levels. On the complete formulation-disjoint hold-out set, Age, W/B, and W/C showed the largest mean absolute SHAP values, indicating that the model assigned the greatest average attribution magnitudes to curing age and the water-related ratio features. Within the GGBS–fly ash blended subset, GGBS showed a larger mean absolute SHAP value than fly ash (1.125 versus 0.722 MPa), while their mean signed SHAP values were +0.250 and −0.603 MPa, respectively. The dependence analysis further showed that the attribution assigned to GGBS generally shifted from negative values at lower contents toward positive values at higher contents, whereas fly ash exhibited a narrower and less consistently ordered contribution pattern. The SCM attribution results were also sensitive to feature representation: removing W/C reversed the ordering of GGBS and fly ash by mean absolute SHAP value, while removing W/B changed both their attribution magnitudes and the average direction assigned to fly ash. These results indicate that SHAP attribution reflects how the fitted model distributes prediction contributions among the selected input variables and that the resulting attribution patterns should be interpreted with regard to the adopted feature representation.

The combined results demonstrate the value of considering not only predictive accuracy but also formulation-level data separation, training-domain coverage, and interpretation sensitivity when machine learning models are developed for SCM-containing concrete. The framework provides a reproducible basis for examining how the composition of available training data affects prediction on blended mixtures. Because the present study used a single public dataset and an internally constructed formulation-disjoint hold-out set, further validation using independently sourced concrete datasets is required to examine the transferability of the observed patterns.

## Figures and Tables

**Table 1:** Descriptive statistics of the cleaned dataset and distribution of mixture types.

**Panel A. Model Input Features and Target Variable**						
**Parameter**	**Unit**	**Mean**	**Standard Deviation**	**Minimum**	**Median**	**Maximum**
Cement	kg/m^3^	274.02	103.20	102.00	261.00	540.00
GGBS	kg/m^3^	72.49	84.07	0.00	24.00	359.40
Fly ash	kg/m^3^	64.28	71.76	0.00	0.00	260.00
Water	kg/m^3^	183.49	21.68	121.75	185.70	247.00
SP	kg/m^3^	6.26	5.75	0.00	6.47	32.20
Coarse aggregate	kg/m^3^	965.99	82.91	708.00	967.00	1145.00
Fine aggregate	kg/m^3^	769.62	79.51	594.00	777.80	992.60
Age	days	44.23	60.94	1.00	28.00	365.00
W/C	–	0.78	0.32	0.27	0.71	1.88
W/B	–	0.47	0.12	0.24	0.47	0.90
Compressive strength	MPa	35.33	15.69	2.33	34.18	82.60
**Panel B. Distribution of mixture types**						
Mixture type		Count			Percentage (%)	
GGBS-only		327			29.54	
Fly ash-only		258			23.31	
GGBS–fly ash blended		289			26.11	
Neither GGBS nor fly ash		233			21.05	
Total		1107			100.00	

**Table 2:** Hyperparameter search spaces of the candidate models.

Model	Key Hyperparameters and Search Spaces
LR	Non-optimized linear reference
DT	max_depth = [3, 16], min_samples_split = [2, 20], min_samples_leaf = [1, 10], splitter = {‘best’, ‘random’}
SVR	kernel = ‘rbf’ (fixed); C = [10^−2^, 10^3^], log scale; gamma = [10^−4^, 1], log scale; epsilon = [10^−3^, 0.5], log scale
MLP	hidden_layer_sizes = {(32,), (64,), (128,), (64, 32), (128, 64)}; activation = {‘relu’, ‘tanh’}; alpha = [10^−6^, 10^−2^], log scale; learning_rate_init = [10^−4^, 10^−2^], log scale; batch_size = {16, 32, 64, 128}
RF	n_estimators = [200, 800], step 50; max_depth = [4, 16]; min_samples_split = [2, 12]; min_samples_leaf = [1, 6]; max_features = {‘sqrt’, ‘log2’, 0.6, 0.8, 1.0}
XGBoost	n_estimators = [200, 800], step 50; max_depth = [3, 8]; learning_rate = [0.01, 0.15], log scale; subsample = [0.7, 1.0]; colsample_bytree = [0.6, 1.0]; min_child_weight = [1, 8]; reg_alpha = [10^−4^, 1], log scale; reg_lambda = [10^−3^, 10], log scale

Note: LR was retained as a non-optimized linear reference. MLP convergence was monitored during nested optimization with an iteration cap of 5000; the selected configuration that still reached this cap was re-fitted with the iteration limit increased to 20,000 while retaining the TPE-selected hyperparameter values.

**Table 3:** Outer-fold predictive performance of the candidate models.

Model	MAE (MPa)	RMSE (MPa)	MAPE (%)	R^2^	RMSE 95% CI (MPa)
XGBoost	3.804 ± 0.631	5.396 ± 0.882	12.276 ± 2.816	0.885 ± 0.040	4.766–6.027
RF	4.045 ± 0.556	5.702 ± 0.883	13.249 ± 2.554	0.873 ± 0.038	5.070–6.334
MLP	4.165 ± 0.792	5.805 ± 1.177	13.550 ± 2.520	0.865 ± 0.057	4.963–6.647
SVR	4.621 ± 0.970	6.530 ± 1.243	15.420 ± 2.803	0.827 ± 0.081	5.641–7.419
DT	5.409 ± 0.738	7.335 ± 0.916	17.216 ± 2.636	0.791 ± 0.046	6.679–7.990
LR	7.774 ± 0.710	10.022 ± 0.849	29.153 ± 3.746	0.609 ± 0.082	9.415–10.630

Note: Values are reported as mean ± standard deviation across the ten outer folds of formulation-grouped nested cross-validation. Approximate 95% confidence intervals for the mean RMSE were calculated using a t-based approximation.

**Table 4:** Hold-out performance of the retained XGBoost model.

Model	Observations	Formulations	MAE (MPa)	RMSE (MPa)	MAPE (%)	R^2^
XGBoost	225	106	3.761	5.095	12.609	0.845

Note: The model was fitted to the complete development set using the final configuration determined before hold-out evaluation.

**Table 5:** Prediction performance of XGBoost under different training-set compositions.

Training Setting	Training Observations	Training Formulations	MAE (MPa)	RMSE (MPa)	MAPE (%)	R^2^
All	882	424	3.894	5.372	12.740	0.808
Non-blended All	656	284	5.478	7.095	16.969	0.666
Blended-only	226	140	3.859	5.346	12.728	0.810
GGBS-only	269	116	6.423	8.505	19.126	0.519
Fly ash-only	201	93	5.554	6.925	16.919	0.681
Neither	186	75	11.412	13.098	30.510	−0.140

Note: Performance metrics were calculated at the observation level on the common blended hold-out subset.

**Table 6:** SHAP contributions of GGBS and fly ash.

Evaluation Population	Feature	Mean Absolute SHAP (MPa)	Mean Signed SHAP (MPa)	Positive SHAP Values (%)
Complete hold-out set	GGBS	1.272	−0.102	33.3
	Fly Ash	0.474	−0.169	51.6
Blended hold-out subset	GGBS	1.125	0.250	52.4
	Fly Ash	0.722	−0.603	12.7

**Table 7:** Sensitivity to alternative feature representations.

**Panel A. Development-Set Predictive Sensitivity**				
**Feature Representation**	**RMSE (MPa)**		**R^2^**	
Full representation	5.258 ± 0.140		0.896 ± 0.006	
Without W/C	5.275 ± 0.165		0.895 ± 0.007	
Without W/B	5.771 ± 0.135		0.875 ± 0.006	
Without W/C and W/B	5.788 ± 0.165		0.874 ± 0.007	
**Panel B. SCM Attribution Sensitivity in the Blended Hold-out Subset**				
**Feature Representation**	**GGBS Mean |SHAP| (MPa)**	**GGBS Mean SHAP (MPa)**	**Fly Ash Mean |SHAP| (MPa)**	**Fly Ash Mean SHAP (MPa)**
Full representation	1.125	+0.250	0.722	−0.603
Without W/C	0.862	+0.060	1.140	−1.121
Without W/B	3.020	+0.996	1.396	+0.924
Without W/C and W/B	2.669	+0.941	1.149	+0.591

Note: Panel A reports mean ± standard deviation across the three cross-validation repetitions.

## Data Availability

The data analyzed in this study are publicly available in the Mendeley Data repository [35].

## Supplementary Materials

The following supporting information can be downloaded at: https://www.mdpi.com/article/10.3390/ma19194215/s1, Table S1: Hyperparameter configurations selected across the ten outer folds of formulation-grouped nested cross-validation; Table S2: Fold-wise predictive performance across the ten outer folds of formulation-grouped nested cross-validation; Table S3: Final XGBoost focused-search settings, search space, and selected hyperparameter values; Table S4: Distribution diagnostics for equal-formulation training-set sampling relative to the common blended hold-out target.
