# Supplementary material for: Interpretable Machine Learning for Compressive Strength Prediction of GGBS–Fly Ash Blended Concrete: Generalization Across Training-Set Compositions and Feature-Attribution Sensitivity
Source: Materials (Basel). 2026 Oct 3;19(19):4215. doi: 10.3390/ma19194215 (PMC13666016; doi:10.3390/ma19194215)
Supplement: Supplementary file 1 [file materials-19-04215-s001.zip › materials-4553378-supplementary.pdf]

Table S1. Hyperparameter configurations selected across the ten outer folds of formulation-grouped nested cross-validation.

Panel A. Decision Tree (DT).

**Outer fold**	**max_depth**	**min_samples_split**	**min_samples_leaf**	**splitter**
1	15	13	3	best
2	11	10	1	random
3	11	7	5	best
4	15	6	4	random
5	15	14	3	best
6	15	6	2	random
7	15	9	3	random
8	10	6	7	best
9	10	11	1	random
10	16	16	2	best

Panel B. Support Vector Regression (SVR).

**Outer fold**	**C**	**gamma**	**epsilon**
1	96.030019	0.06999151	0.013488494
2	292.20677	0.047092712	0.0035774515
3	406.06504	0.040147394	0.0070833228
4	607.24093	0.031808392	0.48731113
5	491.4781	0.038235739	0.012660615
6	649.55922	0.031396197	0.011093559
7	281.96703	0.04733317	0.051143632
8	247.50324	0.050729785	0.0017596039
9	181.18772	0.046993795	0.12866042
10	208.34632	0.044734288	0.2188221

Panel C. Multilayer Perceptron (MLP).

Outer fold	hidden_layer_sizes	activation	alpha	learning_rate_init	batch_size
1	(128, 64)	tanh	4.6362236e-06	0.00010103657	128
2	(128, 64)	tanh	0.003054676	0.00095375788	128
3	(64, 32)	tanh	2.2159612e-05	0.00010419831	32
4	(128, 64)	tanh	9.0744986e-05	0.00092137031	32
5	(128,)	tanh	0.0057272755	0.0097614952	128
6	(128,)	relu	0.00033951708	0.00028328599	64
7	(128, 64)	tanh	0.0018044569	0.0017280533	128
8	(128,)	relu	3.6127017e-05	0.00085091683	128
9	(128,)	relu	5.6041843e-05	0.0011248869	16
10	(128, 64)	tanh	1.0669681e-06	0.0014894428	16

Panel D. Random Forest (RF).

Outer fold	n_estimators	max_depth	min_samples_split	min_samples_leaf	max_features
1	300	15	4	1	0.6

2	400	13	4	1	0.8
3	500	12	3	1	1
4	500	15	2	1	1
5	450	15	2	1	1
6	400	15	2	1	0.8
7	500	16	2	1	1
8	450	14	4	2	0.6
9	600	13	6	1	0.8
10	400	15	4	1	1

Panel E. XGBoost.

Outer fold	n_estimators	max_depth	learning_rate	subsample	colsample_bytree	min_child_weight	reg_alpha	reg_lambda
1	300	7	0.047325618	0.86946327	0.68522522	2	0.05954494	0.41894535
2	550	5	0.029989792	0.72971898	0.60823188	8	0.088517238	0.032392566
3	450	4	0.048111457	0.76136473	0.79760554	1	0.94519961	0.32825056
4	550	3	0.086757438	0.98219941	0.97820867	1	0.95358655	8.8757945
5	750	7	0.018555347	0.85328106	0.69829667	2	0.017257442	0.17749181
6	300	3	0.10392478	0.87975801	0.82926438	1	0.39295023	0.022221993
7	200	5	0.082185486	0.85009014	0.63296588	2	0.00018657015	3.5697313
8	750	5	0.030549921	0.78601377	0.60017451	2	0.006525976	0.087057399
9	400	5	0.11600712	0.81986086	0.72529681	2	0.60016448	0.014081437
10	500	7	0.02989504	0.97227173	0.60181672	1	0.00041228089	3.8067564

Note: LR was retained as a non-optimized linear reference and therefore has no fold-specific hyperparameter configuration. For DT, SVR, RF, and XGBoost, the values shown are those selected independently within each outer-training subset by five-fold formulation-grouped inner cross-validation with 50 TPE trials. For MLP, the values shown are from the 5000-epoch nested retuning. Common MLP settings were solver = adam, early_stopping = False, tol = 1e-4, and n_iter_no_change = 10. The Fold-1 MLP configuration was subsequently refitted with the epoch cap increased from 5000 to 20,000 solely to confirm convergence; its hyperparameters were not re-optimized.

Table S2. Fold-wise predictive performance across the ten outer folds of formulation-grouped nested cross-validation.

Panel A. XGBoost.

Outer fold	MAE (MPa)	RMSE (MPa)	MAPE (%)	R^2
1	2.658	3.700	11.362	0.932
2	4.169	5.710	13.510	0.855
3	3.981	4.793	13.413	0.910
4	4.819	6.809	17.246	0.806
5	3.584	5.553	9.052	0.889
6	4.384	5.915	13.437	0.851
7	3.841	5.631	13.220	0.884
8	3.725	6.061	9.594	0.876
9	3.891	5.320	14.104	0.909
10	2.985	4.468	7.823	0.937
Mean ± SD	3.804 ± 0.631	5.396 ± 0.882	12.276 ± 2.816	0.885 ± 0.040

Panel B. Random Forest (RF).

Outer fold	MAE (MPa)	RMSE (MPa)	MAPE (%)	R^2
1	3.043	4.047	13.019	0.919
2	4.226	5.916	14.333	0.844
3	4.313	5.272	13.818	0.891
4	4.836	6.873	16.964	0.802
5	4.180	6.264	11.300	0.858
6	3.959	5.265	13.011	0.882
7	4.226	6.354	15.378	0.853
8	4.285	6.587	11.419	0.854
9	4.273	5.722	15.252	0.895
10	3.108	4.719	7.996	0.929
Mean ± SD	4.045 ± 0.556	5.702 ± 0.883	13.249 ± 2.554	0.873 ± 0.038

Panel C. Multilayer Perceptron (MLP).

Outer fold	MAE (MPa)	RMSE (MPa)	MAPE (%)	R^2
1	2.806	3.739	13.071	0.931
2	4.532	6.488	13.506	0.813
3	4.307	5.934	13.404	0.862
4	5.538	7.765	19.482	0.747
5	3.411	4.570	10.156	0.925
6	4.315	5.662	13.572	0.864
7	4.257	6.054	14.024	0.866
8	5.009	6.834	15.010	0.843
9	3.545	4.785	12.156	0.926
10	3.927	6.215	11.123	0.877
Mean ± SD	4.165 ± 0.792	5.805 ± 1.177	13.550 ± 2.520	0.865 ± 0.057

Panel D. Support Vector Regression (SVR).

Outer fold	MAE (MPa)	RMSE (MPa)	MAPE (%)	R^2
1	3.677	5.106	16.397	0.871

2	5.095	7.212	15.830	0.769
3	4.098	6.279	12.646	0.845
4	5.003	7.040	17.587	0.792
5	4.156	5.733	13.598	0.881
6	6.889	9.218	19.183	0.639
7	5.179	7.520	18.074	0.794
8	4.292	6.097	12.952	0.875
9	4.179	5.815	17.309	0.891
10	3.640	5.285	10.622	0.911
Mean ± SD	4.621 ± 0.970	6.530 ± 1.243	15.420 ± 2.803	0.827 ± 0.081

Panel E. Decision Tree (DT).

Outer fold	MAE (MPa)	RMSE (MPa)	MAPE (%)	R^2
1	4.223	6.070	15.578	0.818
2	5.898	7.763	18.897	0.732
3	4.616	6.037	15.183	0.857
4	5.292	7.333	18.726	0.775
5	6.223	8.268	16.825	0.753
6	5.737	7.246	17.812	0.777
7	5.870	8.154	19.299	0.758
8	5.546	8.223	14.904	0.773
9	6.238	8.038	21.953	0.792
10	4.448	6.214	12.976	0.877
Mean ± SD	5.409 ± 0.738	7.335 ± 0.916	17.216 ± 2.636	0.791 ± 0.046

Panel F. Linear Regression (LR).

Outer fold	MAE (MPa)	RMSE (MPa)	MAPE (%)	R^2
1	6.991	8.704	34.257	0.625
2	7.551	9.901	28.598	0.564
3	8.018	10.303	27.711	0.584
4	8.393	10.698	35.420	0.520
5	8.030	10.352	25.143	0.613
6	8.588	11.063	30.623	0.481
7	7.462	9.759	28.290	0.653
8	8.560	11.041	29.057	0.590
9	7.783	9.724	29.560	0.696
10	6.362	8.678	22.876	0.761
Mean ± SD	7.774 ± 0.710	10.022 ± 0.849	29.153 ± 3.746	0.609 ± 0.082

Note: Metrics were calculated separately on each held-out outer fold at the observation level. The mean ± SD rows reproduce the fold-wise summaries reported in Table 3 of the revised manuscript. For DT, SVR, RF, XGBoost, and LR, values are from the formulation-grouped nested cross-validation results. For MLP, values are from the updated ten-fold results after Fold-1 convergence confirmation; only the Fold-1 outer predictions were replaced using the same previously selected hyperparameters with the epoch cap increased to 20,000, without re-optimization.

Table S3. Final XGBoost focused-search settings, search space, and selected hyperparameter values.

Following candidate-model comparison, the final XGBoost configuration was determined on the complete development set using three repetitions of five-fold formulation-grouped cross-validation (seeds 42, 2026, and 3407). The final focused search comprised 250 TPE trials, with the mean pooled out-of-fold RMSE across the three repetitions used as the optimization objective.

**Hyperparameter**	**Selected value**	**Final focused search space**
n_estimators	1400	700–1600, step 50
max_depth	4	3–7
learning_rate	0.019168629989610615	0.004–0.03, log scale
subsample	0.7604483026384505	0.55–0.90
colsample_bytree	0.6757950689877155	0.55–0.90
min_child_weight	0	0–6
gamma	0.7389626005949499	0–1
reg_alpha	0.0033684538080148148	10^{-5}–0.05, log scale
reg_lambda	0.00016361889518936266	10^{-5}–0.1, log scale

Note: This final XGBoost search space was defined separately from the candidate-model comparison search space reported in Table 2. The formulation-disjoint hold-out set was not used for hyperparameter selection.

Table S4. Distribution diagnostics for equal-formulation training-set sampling relative to the common blended hold-out target.

Panel A. Mean absolute standardized mean differences (|SMD|).

Formulations	Training setting	Mean training observations	Age	Strength	Total binder	W/B
25	All	51.92	0.245	0.175	0.396	0.480
25	Non-blended All	56.32	0.321	0.230	0.611	0.750
25	Blended-only	40.20	0.080	0.159	0.198	0.252
25	GGBS-only	59.94	0.359	0.176	0.245	0.408
25	Fly-ash-only	53.80	0.162	0.313	1.092	0.850
25	Neither	63.80	0.411	0.285	0.922	1.116
50	All	102.42	0.261	0.128	0.408	0.520
50	Non-blended All	112.38	0.332	0.160	0.595	0.733
50	Blended-only	81.86	0.067	0.131	0.151	0.203
50	GGBS-only	116.62	0.366	0.124	0.201	0.398
50	Fly-ash-only	107.36	0.167	0.339	1.114	0.900
50	Neither	125.54	0.412	0.263	0.898	1.093
75	All	153.16	0.265	0.103	0.401	0.507
75	Non-blended All	172.22	0.333	0.135	0.589	0.720
75	Blended-only	122.58	0.062	0.114	0.125	0.183
75	GGBS-only	174.62	0.362	0.133	0.144	0.366
75	Fly-ash-only	160.44	0.169	0.344	1.108	0.910
75	Neither	186.00	0.404	0.264	0.898	1.093

Panel B. Mean fraction of target observations outside the sampled training range (%).

Formulations	Training setting	Age	Strength	Total binder	W/B
25	All	0.4	1.7	9.4	3.7
25	Non-blended All	0.1	1.4	13.8	3.8
25	Blended-only	0.3	5.3	15.4	5.7
25	GGBS-only	0.5	0.1	4.3	1.1
25	Fly-ash-only	0.0	5.9	18.3	13.4
25	Neither	0.0	1.1	25.4	1.3
50	All	0.0	0.6	3.5	0.0
50	Non-blended All	0.0	0.2	7.0	0.0
50	Blended-only	0.0	2.3	9.6	1.7
50	GGBS-only	0.0	0.0	0.6	0.0
50	Fly-ash-only	0.0	4.3	11.7	4.3
50	Neither	0.0	0.5	25.4	0.0
75	All	0.0	0.2	1.0	0.0
75	Non-blended All	0.0	0.1	3.3	0.0
75	Blended-only	0.0	1.7	8.2	0.7
75	GGBS-only	0.0	0.0	0.2	0.0
75	Fly-ash-only	0.0	3.7	8.8	0.0
75	Neither	0.0	0.0	25.4	0.0

Note: Values are means across 50 formulation-level sampling repetitions at each formulation count. For Panel A, the standardized mean difference (SMD) compares the sampled training

subset with the common GGBS–fly ash blended hold-out target; absolute values are reported. Panel B reports the mean percentage of target observations falling outside the sampled training range for each variable. These distribution diagnostics were calculated after sampling and were not used to select training formulations; the procedure controlled formulation count rather than matching covariate distributions. Mean training observation counts differ because all observations associated with a selected formulation were retained together.
